# Clinical utility and psychometric properties of tools for early detection of developmental concerns and disability in young children: A scoping review

**DOI:** 10.1111/dmcn.16076

**Published:** 2024-09-16

**Authors:** Andrea Burgess, Carly Luke, Michelle Jackman, Jane Wotherspoon, Koa Whittingham, Katherine Benfer, Sarah Goodman, Rebecca Caesar, Tiffney Nesakumar, Samudragupta Bora, David Honeyman, Danielle Copplin, Sarah Reedman, John Cairney, Natasha Reid, Leanne Sakzewski, Roslyn N. Boyd

**Affiliations:** ^1^ Queensland Cerebral Palsy and Rehabilitation Research Centre, Faculty of Medicine The University of Queensland Brisbane QLD Australia; ^2^ John Hunter Children's Hospital Newcastle NSW Australia; ^3^ Women's and Children's Service Sunshine Coast University Hospital QLD Australia; ^4^ Health Services Research Center, University Hospitals Research and Education Institute; Department of Pediatrics, University Hospitals Rainbow Babies and Children's Hospital Case Western Reserve University School of Medicine Cleveland OH USA; ^5^ Mater Research Institute, Faculty of Medicine & School of Psychology, Faculty of Health and Behavioural Sciences The University of Queensland QLD Australia; ^6^ School of Human Movement, Faculty of Health and Behavioural Sciences The University of Queensland Brisbane QLD Australia; ^7^ Faculty of Medicine Library The University of Queensland Brisbane QLD Australia; ^8^ Child Health Research Centre The University of Queensland QLD Australia

## Abstract

**Aim:**

To explore the clinical utility and psychometric properties of standardized tools for the early detection of developmental concerns or disability in young children.

**Method:**

Systematic reviews and clinical practice guidelines containing psychometric data on tools appropriate for use with children from birth to 5 years 11 months were searched for in MEDLINE, CINAHL, Embase, and PsycINFO for the years 2000 to 2023, with no language restrictions.

**Results:**

Eighty‐six systematic reviews and six clinical practice guidelines guided identification of tools. A total of 246 tools were identified across domains of neurological, motor, cognition, communication/language, social–emotional, sensory processing, and/or specific diagnostic conditions of attention‐deficit/hyperactivity disorder, autism spectrum disorder, cerebral palsy, developmental coordination disorder, and fetal alcohol spectrum disorder. After critical evaluation, 67 tools were included in the recommendations. Recommendations for screening and diagnostic assessment tools were based on best available evidence for predictive and discriminative validity, diagnostic accuracy, together with consideration of resource use and accessibility.

**Interpretation:**

This comprehensive scoping review provides recommendations on the best tools for primary care, medical, allied health professionals, nursing, and other health workers to detect and identify developmental concerns or disability in young children using evidence‐based tools.

## Abstract 2

Plain language summary: https://onlinelibrary.wiley.com/doi/10.1111/dmcn.16140

**Abbreviations**
ABAS‐3: Adaptive Behavior Assessment System, Third Edition
ADI‐R: Revised Autism Diagnostic Interview
ADOS‐2: Autism Diagnostic Observational Schedule, Second Edition
AIMS: Alberta Infant Motor Scale
ASD: autism spectrum disorder
ASQ‐3: Ages & Stages Questionnaires, Third Edition
ASQ‐TRAK: Ages & Stages Questionnaires ‐ Talking About Raising Aboriginal Kids
BADDS: Brown Attention Deficit Disorder Symptom Assessment Scale
BASC: Behaviour Assessment System for Children
Bayley‐III: Bayley Scales of Infant and Toddler Development, Third Edition
BDI‐2: Battelle Developmental Inventory, Second Edition
BITSEA: Brief Infant Toddler Social and Emotional Assessment
BOT‐2: Bruininks‐Oseretsky Test of Motor Proficiency
BRIEF‐P: Behavior Rating Index of Executive Function–Preschool
CARS: Childhood Autism Rating Scale
CBCL: Child Behaviour Checklist
CELF‐5: Clinical Evaluation of Language Fundamentals, Fifth Edition
Conners EC: Conners Early Childhood Assessment
CSBS: Communication and Symbolic Behavior Scales
DAS‐II: Differential Ability Scales, Second Edition
DCD: developmental coordination disorder
FASD: fetal alcohol spectrum disorder
GMA: Prechtl's General Movements Assessment
GMFM: Gross Motor Function Measure
HAI: Hand Assessment for Infants
HATS: Hearing and Talking Scale
HINE: Hammersmith Infant Neurological Examination
ITSEA: Infant Toddler Social and Emotional Assessment
MABC‐2: Motor Assessment Battery for Children, Second Edition
M‐CHAT‐R/F: Modified Checklist for Autism in Toddlers, with Follow‐up
MSEL: Mullen Scales of Early Learning
PDMS‐2: Peabody Developmental Motor Scales, Second Edition
PEDI: Pediatric Evaluation of Disability Inventory
PEDI‐CAT: Pediatric Evaluation of Disability Inventory ‐ Computer‐Adaptive Test
PEDS: Parents' Evaluation of Developmental Status
PLUM: Parents' Evaluation of Listening and Understanding Measure
PSEQ‐H: Participation and Sensory Environment Questionnaire‐Home
SACS‐PR: Social Attention and Communication Surveillance Preschool
SACS‐R: Revised Social Attention and Communication Surveillance
SDQ: Strengths and Difficulties Questionnaire
TGMD‐2: Test of Gross Motor Development, Second Edition
VABS: Vineland Adaptive Behavior Scales
WPPSI‐IV: Wechsler Preschool and Primary Scale of Intelligence, Fourth Edition

**What this paper adds**

- Recommendations are provided for the most evidence‐based and accessible screening and diagnostic assessment tools.
- The recommended multi‐domain screening tool is the Ages & Stages Questionnaires.
- Recommended multi‐domain developmental assessment tools are the Bayley Scales of Infant and Toddler Development, the Battelle Developmental Inventory, and the Mullen Scales of Early Learning.
- Recommended diagnostic assessment tools for cerebral palsy are Prechtl's Qualitative Assessment of General Movements and the Hammersmith Infant Neurological Examination.
- The recommended diagnostic screening tool for autism is the revised version of the Social Attention and Communication Surveillance.

Neurodevelopmental disorders are a group of conditions that manifest early in development, often before a child enters school. They can be characterized by developmental deficits, and they produce impairments of personal, social, academic, or occupational functioning (Diagnostic Criteria of Neurodevelopmental Disorders from the Diagnostic and Statistical Manual of Mental Disorders, Fifth Edition, Text Revision, DSM‐5‐TR).^1^ Neurodevelopmental disorders considered in this review are those most commonly diagnosed, including intellectual developmental disorder (intellectual disability), global developmental delay, communication disorders, autism spectrum disorder (ASD), attention‐deficit/hyperactivity disorder (ADHD), cerebral palsy (CP), developmental coordination disorder (DCD), and fetal alcohol spectrum disorder (FASD).^1^

Neurodevelopmental disorders frequently co‐occur with one another.^1^ Intellectual disability is the most commonly reported disability among children (e.g. 58% of Australian children who have a disability have an intellectual disability).^2^ CP is the most common physical disability in childhood (1 in 700 children).^3^, ^4^ The prevalence of autism is approximately 1 in 100 children.^5^ Definitions of neurodevelopmental disorders used in this review are those of the DSM‐5‐TR.^1^

Early recognition of a developmental concern or disability is crucial for children and their families—helping families and carers to access appropriate supports and services that can facilitate their child's development and learning.^6^ Timely recognition of developmental concerns or disability in young children optimizes outcomes.^6^ Screening and assessment tools can support primary healthcare professionals to identify developmental concerns or disability at an early age, facilitating timely referral and access to intervention and supports. Screening tools are those that are used to identify children who are developing typically or who are at higher risk of developmental delays and are used to identify children who require more in‐depth evaluation. Screening tools can be administered by a range of community and early childhood professionals. Diagnostic/assessment tools provide in‐depth assessment of a child to determine the nature and extent of a developmental delay or to aid in diagnosis and are administered by appropriately trained practitioners. Clinical practice guidelines highlight the importance of comprehensive multidisciplinary assessment for diagnosing neurodevelopmental conditions. A single screening or assessment tool cannot be used in isolation for diagnosis.

The aim of this scoping review was to provide primary healthcare professionals, namely general practitioners, paediatricians, nurses and midwives, allied health professionals (psychology, occupational therapy, physiotherapy, speech pathology), and community health workers, with information to inform the selection of the best evidence‐based tools/assessments for the early detection of developmental concerns or disability in young children from birth to 5 years 11 months of age. Specifically, the review had the following aims: (1) to identify available assessments/tools for screening and diagnosis of children from birth to 5 years 11 months; (2) to provide recommendations on the most effective tools on the basis of available evidence for predictive and discriminative validity, diagnostic accuracy, and clinical utility.

## METHOD

### Searches

This scoping review of tools for the early detection of developmental delay or disability followed best practice guidelines as provided by the JBI Scoping Review network (https://jbi.global/scoping‐review‐network/resources).^7^ The process involved clearly defining the purpose and scope of the review; searching, identifying, and selecting studies; extraction/charting and categorizing data; and reporting and summarizing the results. The review was registered on Figshare (https://doi.org/10.6084/m9.figshare.21708248.v1) on 13th December 2022.^8^

Multiple approaches were used to search as broadly as possible and entailed the following: (1) a comprehensive search of relevant systematic reviews across four databases; (2) a comprehensive desktop search of grey literature sources (government and non‐government, for example national and international clinical practice guidelines); and (3) specific searches on validation studies of tools identified in government guidelines and clinical practice guidelines that were not contained in the systematic reviews.

The comprehensive search of systematic reviews was undertaken across four electronic databases: MEDLINE ALL (Ovid), CINAHL (EBSCO host), Embase (Embase.com), and PsycInfo (APA PsycNet). The search was limited to the years 2000 up to July 2023, and was not limited to the English language. The search strategies were developed by the first (AB) and second (CL) authors with the research librarian (DH). The search strategy used Medical Subject Headings (MeSH) terms and keywords for ‘children’; ‘disability’, ‘neurodevelopmental disorders’ or specific diagnoses; ‘assessments’ or ‘tools’; and ‘psychometrics’. To limit the search to systematic reviews, predefined methodological search filters were used. The MEDLINE ALL search was combined with the Search Strategy Used to Create the PubMed Systematic Reviews Filter (National Library of Medicine, 2018, adapted for MEDLINE Ovid), the CINAHL and Embase searches were combined with the Scottish Intercollegiate Guidelines Network systematic review search filters, and the methodology limit ‘systematic review’ was selected in PsycInfo. The search strategy is reported in Appendix S1. The search was initially performed on the 3rd August 2022 and updated on the 19th July 2023.

Systematic reviews were included if they met the following inclusion criteria: (1) contained tools/assessments that had published validity for children from birth to 5 years 11 months; (2) examined psychometric properties of tools (predictive, discriminative, or evaluative); (3) contained tools that assessed domains of motor (gross or fine), cognition, language/communication, behaviour, or social–emotional, and/or other neurodevelopmental outcomes; (4) contained tools used for diagnosing specific disabilities such as CP, autism, intellectual disability, DCD, and FASD; and (5) contained tools/assessments that could be parent‐reported or patient‐reported outcome measures and/or standardized observational tools with normative data.

Systematic reviews were excluded if they met the following inclusion criteria: (1) were outside the age range (i.e. 6 + years); (2) did not examine tools that primarily assessed domains of motor (gross or fine), cognition, language/communication, sensory–motor, behaviour, or social–emotional or other neurodevelopmental outcomes; and (3) examined tools not available for use by primary healthcare professionals, allied health professionals, and community health practitioners (e.g. genetic testing, magnetic resonance imaging, tests exclusively performed in the neonatal setting).

Grey literature searches were performed by one author (AB) with guidance from the research librarian (DH). Google searches used a phrase (e.g. ‘diagnosis of autism in children’). The first four pages of search results were examined for relevant information. Relevant information was recorded in table form (Appendix S1). Targeted reference scanning and citation tracking of key papers were performed. Articles describing the tool were included if more than 90% of the study population were within the age range birth to 5 years 11 months.

### Study selection of systematic reviews

Titles and abstracts of systematic reviews were independently screened by two reviewers (AB and CL) using Covidence. In cases of disagreement, a discussion was held and consensus reached. Full‐text screening was performed by one reviewer (AB). The authors reviewed and confirmed tools and performed data extraction in teams by domain. As review teams performed data extraction, any full‐text papers judged not to meet inclusion criteria were highlighted to the one reviewer (AB), discussed, and consensus reached about inclusion.

### Data charting

Papers were categorized into tools that assessed domains of child development or tools related to specific diagnoses. Domains were neurological, cognition, communication and language, motor, sensory processing, social–emotional, and multi‐domain tools and assessments. Diagnostic classifications were ASD, ADHD, FASD, CP, and DCD. Review teams comprised experts in each domain of child development or diagnoses to perform independent review and data charting.

Data charting was performed for tools/assessments from the included systematic reviews and clinical practice guidelines about psychometric properties and clinical utility (Appendix S2). Data were charted from papers in order of publication recency to avoid duplication of data from previous reviews. Systematic review summaries that used COnsensus‐based Standards for the selection of health Measurement INstruments (COSMIN)^9^ were used when available. COSMIN guidelines^9^ were followed when reporting on measurement properties and quality of studies. Identified tools/assessments were ranked within domains for recommendation on the basis of predictive and discriminative validity and clinical utility by the review team of experts.

## RESULTS

### Search results

Figure S1 shows the Preferred Reporting Items for Systematic reviews and Meta‐Analyses (PRISMA) flowchart for the systematic review search. Eighty‐six systematic reviews (Appendix S5), four national clinical guidelines,^10^, ^11^, ^12^, ^13^ and two international clinical guidelines^14^, ^15^ were included in this review. A total of 133 additional papers were sourced to provide further information on specific measures. A total of 246 tools were identified, of which 67 were appropriate for children aged from birth to 5 years 11 months, had published validity and reliability, and were available for use (Table 1; Appendix S2 and S3). Some tools assessed multiple domains of development, others were specific to a domain or diagnosis (e.g. autism), while yet other tools could be used across different domains and diagnoses (such as ADHD, behaviour, and social–emotional domain). Four tools were identified that had been specifically validated for Australian Aboriginal and Torres Strait Islander children.^16^, ^17^, ^18^, ^19^

**TABLE 1:** Summary of recommended tools by domain and age for screening, assessment, and diagnosis of developmental concerns and disability.

Domain	Screening 0–2 years	Assessment 0–2 years	Screening 2–5 years	Assessment 2–5 years
Multi‐domain	Ages & Stages Questionnaires‐3^a^, ^b^ ^,^ ^29^ Ages & Stages Questionnaires ‐ Talking About Raising Aboriginal Kids^a^, ^c^ ^,^ ^19^ Parents Evaluation of Developmental Status^a^, ^b^,^34^	**Developmental:** Bayley Scales of Infant and Toddler Development, Third Edition^40^ Battelle Developmental Inventory, Second Edition^41^ Mullen Scales of Early Learning^42^ **Adaptive functioning:** Pediatric Evaluation of Disability Inventory^53^ Pediatric Evaluation of Disability Inventory ‐ Computer‐Adaptive Test^54^ Vineland Adaptive Behaviour Scales^55^ Adaptive Behavior Assessment system^56^	Ages & Stages Questionnaires‐3^a^, ^b^ ^,^ ^29^ Ages & Stages Questionnaires ‐ Talking About Raising Aboriginal Kids^a^, ^c^ ^,^ ^19^ Parents Evaluation of Developmental Status^a^, ^b^ ^,^ ^34^ Parent Report of Children's Abilities ‐ Revised (at 2 years)^a^, ^b^, ^d^ ^,^ ^36^	**Developmental:** Bayley Scales of Infant and Toddler Development, Third Edition^40^ Battelle Developmental Inventory, Second Edition^41^ Mullen Scales of Early Learning^42^ **Adaptive functioning:** Pediatric Evaluation of Disability Inventory^53^ Pediatric Evaluation of Disability Inventory ‐ Computer‐Adaptive Test^54^ Vineland Adaptive Behavior Scales^55^ Adaptive Behavior Assessment system^56^
Neurological	Prechtl's Qualitative Assessment of General Movements (0–5 months of age) and Motor Optimality Score (3–5 months)^a^, ^b^ ^,^ ^60^	Hammersmith Infant Neurological Examination (2 months–2 years)^61^ Hand Assessment of Infants (3–12 months)^62^		Neurological Sensory Motor Developmental Assessment (from 2 years of age)^63^
Motor	Alberta Infant Motor Scale^85^	Peabody Developmental Motor Scales, Second Edition^86^	Developmental Coordination Disorder Questionnaire^a^, ^d^ ^,^ ^163^ Little Developmental Coordination Disorder Questionnaire^a^ ^,^ ^164^	Peabody Developmental Motor Scales, Second Edition^86^ Movement Assessment Battery for Children, Second Edition (3 years +)^87^ Test of Gross Motor Development, Second Edition^88^ Bruininks‐Oseretsky Test of Motor Proficiency (4 + years)^89^
Fine motor		Hand Assessment of Infants (3–12 months)^62^ Relevant fine‐motor domain of multi‐domain/ motor assessment tools		Relevant multidomain and motor assessment: e.g. Peabody Developmental Motor Scales, Second Edition;^86^ Motor Assessment Battery for Children, Second Edition;^87^ Mullen Scales of Early Learning;^42^ Battelle Developmental Inventory, Second and Third Edition;^41^ Bruininks‐Oseretsky Test of Motor Proficiency^89^
Cognition		Bayley Scales of Infant and Toddler Development, Third/Fourth Edition (between 18 months and 40 months)^40^ Griffiths Mental Development Scales^52^		Wechsler Preschool and Primary Scale of Intelligence, Fourth Edition^105^ Stanford‐Binet Intelligence Scales^176^ Differential Ability Scales‐II^177^ Developmental NEuroPSYchological Assessment^176^ Revised Leiter International Performance Scale^106^
Communication/language	Communication and Symbolic Behavior Scales (Infant‐Toddler) (6–24 months)^a^ ^,^ ^108^ Hearing and Talking Scale^b^, ^c^, ^d^ ^,^ ^17^ Parents' Evaluation of Listening and Understanding Measure^b^, ^c^, ^d^ ^,^ ^16^	Preschool Language Scales, Fifth Edition^114^	Communication and Symbolic Behavior Scales (Infant‐Toddler) (6–72 months for children with significant delay)^a^ ^,^ ^108^ Hearing and Talking Scale^b^, ^c^, ^d^ ^,^ ^17^ Parents' Evaluation of Listening and Understanding Measure^b^, ^c^, ^d^ ^,^ ^16^	**Speech:** Diagnostic Evaluation of Articulation and Phonology^113^ Dynamic Evaluation of Motor Speech Skill^111^ **Language:** Preschool Language Scales, Fifth Edition^114^ Clinical Evaluation of Language Fundamentals Preschool, Second Edition (3–7 years)^115^ Clinical Evaluation of Language Fundamentals, Fifth Edition (5+ years)^115^
Social–emotional	Brief Infant Toddler Social and Emotional Assessment^116^ The Child Behaviour Checklist 1.5/5^b^ ^,^ ^123^ Ages & Stages Questionnaires: Social–Emotional, Second Edition^118^	Infant Toddler Social and Emotional Assessment (1–3 years)^117^ Greenspan Social–Emotional Growth Chart (0–3.5 years)^124^	Strengths & Difficulties Questionnaire^a^, ^b^, ^d^ ^,^ ^119^ Ages & Stages Questionnaires: Social–Emotional, Second Edition^118^	The Behaviour Assessment System for Children (2 years +)^122^ The Child Behaviour Checklist^123^ Greenspan Social–Emotional Growth Chart (0–3.5 years)^124^ Infant Toddler Social and Emotional Assessment (1–3 years)^117^ The Indigenous Child‐Initiated Pretend Play Assessment with its component of the Indigenous Play Partner Scale^c^ ^,^ ^18^
Sensory processing		Test of Sensory Function in Infants – 4 to 18 months^96^ Infant/Toddler Sensory Profile 2^177^		Participation and Sensory Environment Questionnaire‐Home (2–7 years)^178,179^ Child Sensory Profile‐2 (3–14 years)^98^ Sensory Processing Measure‐Preschool (2–5 years)^180^
**Diagnostic group**	**Screening 0–2 years**	**Assessment 0–2 years**	**Screening 2–5 years**	**Assessment 2–5 years**
Cerebral palsy	Prechtl's Qualitative Assessment of General Movements (0–5 months)^a^, ^b^ ^,^ ^60^	Hammersmith Infant Neurological Examination (2–24 months)^61^ Developmental Assessment of Young Children^127^ Hand Assessment of Infants^62^	Gross Motor Function Classification System^129^ Manual Ability Classification System^130^ Communication Function Classification System^131^	Gross Motor Function Measure^132^
Attention‐deficit/hyperactivity disorder	Child Behaviour Checklist 1.5/5^b^ ^,^ ^123^ Strengths and Difficulties Questionnaire^a^, ^b^, ^d^ ^,^ ^119^	Behavior Assessment System for Children^122^ Behavior Rating Index of Executive Function–Preschool (BRIEF‐P)^157^ Brown Attention Deficit Disorder Symptom Assessment Scale^158^	Strengths and Difficulties Questionnaire^a^, ^b^, ^d^ ^,^ ^119^ Child Behaviour Checklist 1.5/5^a^, ^b^ ^,^ ^123^	**Measures of attention and behaviour:** Behavior Assessment System for Children, Third Edition^122^ Conners Early Childhood Ratings Scales^159^ **Focus on the executive function:** BRIEF‐P^157^ Brown Attention Deficit Disorder Symptom Assessment Scale^158^
Autism spectrum disorder	Revised Social Attention and Communication Surveillance^a^, ^b^ ^,^ ^135^ ASDetect smartphone app^a^, ^b^, ^d^ ^,^ ^137^ Revised Modified Checklist for Autism in Toddlers with Follow‐up^a^, ^b^, ^d^ ^,^ ^136^	The Autism Diagnostic Observational Schedule, Second Edition (12 months +)^150^ Childhood Autism Rating Scale^151^ Autism Observation Scale for Infants (6–36 months)^153^	Social Attention and Communication Surveillance Preschool^a^, ^b^ ^,^ ^135^ Revised Modified Checklist for Autism in Toddlers with Follow‐up^a^, ^b^, ^d^ ^,^ ^136^	Autism Diagnostic Observational Schedule, Second Edition^150^ Childhood Autism Rating Scale^151^ Revised Autism Diagnostic Interview^152^
Developmental coordination disorder			Developmental Coordination Disorder Questionnaire^a^, ^b^, ^d^ ^,^ ^163^ Little Developmental Coordination Disorder Questionnaire^a^, ^b^ ^,^ ^165^	Movement Assessment Battery for Children, Second Edition^87^
Fetal alcohol spectrum disorder	Routinely gathering information on prenatal alcohol exposure when taking developmental history^11^, ^166^	For a diagnosis of fetal alcohol spectrum disorder three criteria must be considered: (1) prenatal alcohol use (AUDIT‐C)^167^ and other exposures, (2) neurodevelopmental impairment, and (3) sentinel facial features	Routinely gathering information on prenatal alcohol exposure when taking developmental history^11^, ^166^	For a diagnosis of fetal alcohol spectrum disorder, three criteria must be considered: (1) prenatal alcohol use (AUDIT‐C)^167^ and other exposures, (2) neurodevelopmental impairment, and (3) sentinel facial features

^a^ Available for use by all health professionals (general practitioners, nurses, Indigenous health workers, allied health professionals).

^b^ Telehealth‐friendly.

^c^ Culturally adapted for Australian Aboriginal and Torres Strait Islander children.

^d^ Freely available (no cost).

### Multi‐domain tools

Multi‐domain assessment tools can be used to screen or assess a child across multiple developmental domains (e.g. cognition, motor skills, communication, etc.) or multiple functional/adaptive domains (e.g. self‐care, mobility, socialization).

#### Multi‐domain developmental screening tools

There was extensive psychometric evidence^20^, ^21^, ^22^, ^23^, ^24^, ^25^, ^26^, ^27^ for the Ages & Stages Questionnaires, Third Edition (ASQ‐3).^28^, ^29^ The ASQ was recently recommended as the most appropriate multidimensional screening tool of child development.^21^, ^30^ The ASQ‐3 aims to identify children with concerns across cognition, motor, communication, and personal–social domains from 1 month to 5 years 6 months (1–66 months) of age. The ASQ is the most widely used developmental screening questionnaire in follow‐up cohort studies.^25^ A recent systematic review^23^ found that, by using a threshold of more than 2 standard deviations (SD) below the mean, the ASQ had pooled sensitivity of 0.77 (95% confidence interval [CI] 0.64–0.86) and specificity of 0.81 (95% CI 0.75–0.86) to diagnose any developmental delay, and a sensitivity of 0.84 (95% CI 0.75–0.90) and specificity of 0.77 (95% CI 0.71–0.82) to diagnose severe developmental delay in children aged 12 to 60 months.^23^ The ASQ is easy to use and can be administered through telehealth with parents/caregivers. The ASQ‐3 is currently available in 11 languages. Cultural validation of the ASQ for Indigenous Australian children has led to the development of the Ages & Stages Questionnaires ‐ Talking About Raising Aboriginal Kids (ASQ‐TRAK).^31^, ^32^

There was evidence available for the Parents' Evaluation of Developmental Status (PEDS;^33^ the revised version of the PEDS; PEDS‐Developmental Milestones^34^). The PEDS uses 10 questions to elicit parents' concerns in a range of developmental areas. If any concerns arise, then use of a standardized screener such as the ASQ‐3^29^ or PEDS‐Developmental Milestones is recommended.^34^, ^35^ The PEDS‐Developmental Milestones provides monitoring of development, using milestone checklists with clear cut‐offs for ‘typical’ or ‘concerns’ in development.^34^ There was limited evidence available about the Denver Developmental Screening Test‐II; a recent systematic review found it did not meet standards of psychometric evidence.^21^

The revised version of the Parent Report of Children's Abilities is a norm‐referenced assessment of cognitive and language development at 2 years of age.^36^, ^37^ It is a parent‐completed questionnaire, freely available online. Concurrent validity testing with the Bayley Scales of Infant and Toddler Development, Third Edition (Bayley‐III) successfully predicted cases of cognitive delay and language delay, with receiver operating characteristic curves ranging from 0.83 to 0.97.^38^ The revised version of the Parent Report of Children's Abilities is recommended by the National Institute for Health and Care Excellence (NICE) for screening 2‐year‐old children born preterm.^39^

#### Multi‐domain developmental assessment tools

Multi‐domain developmental assessment tools are designed to identify developmental delay. A 2020 systematic review^21^ of multi‐domain tools found three developmental assessment tools with good to excellent psychometric properties: Bayley‐III,^40^ the Battelle Developmental Inventory, Second Edition (BDI‐2),^41^ and the Mullen Scales of Early Learning (MSEL)^42^ in order of psychometric quality (Appendix S2). The Bayley‐III identifies children and infants who are not meeting developmental milestones across cognitive, language, and motor domains.^40^ The Bayley‐III has predictive validity for CP at 4 years: for example, at 2 years, less than 1SD, it has a sensitivity of 0.83 and specificity of 0.94; at less than 2SD, its sensitivity is 0.67 and specificity 1.0.^43^, ^44^ The BDI‐2 covers domains of cognition, motor, communication, personal–social, and adaptive skills up to 95 months of age.^42^, ^45^ Both the Bayley‐III and the BDI‐2 have good psychometric evidence.^21^, ^46^ The Bayley‐4 and the BDI‐3 are now available; however, no published information of these new versions was available in systematic reviews. The MSEL assesses children aged 0 to 68 months for expressive language, receptive language, gross motor, fine motor, and visual reception.^42^ The MSEL provides an early learning score (all domains except the gross motor scale) and has validity for cognitive assessment.^47^, ^48^, ^49^ The MSEL has been used for the prospective assessment of infant siblings of children with autism.^50^, ^51^ The MSEL has separate scales for visual reception (non‐verbal abilities) and receptive and expressive language;^42^ it detects asymmetry in developing abilities. The revised version of the Griffiths Mental Developmental Scales^52^ was found to have unsatisfactory psychometric properties.^21^

#### Multi‐domain functional/adaptive assessment tools

Multi‐domain functional/adaptive assessment tools are used to measure functional performance and adaptive behaviour across several domains. The Pediatric Evaluation of Disability Inventory (PEDI),^53^ Pediatric Evaluation of Disability Inventory ‐ Computer‐Adaptive Test (PEDI‐CAT),^54^ the Vineland Adaptive Behavior Scales (VABS‐2/3),^55^ and the Adaptive Behavior Assessment System, Third Edition (ABAS‐3)^56^ are standardized multi‐domain assessments based on large normative samples, with the primary purpose of detecting functional delay. The PEDI is a paper questionnaire of self‐care, mobility, and social function for children aged 6 months to 7 years 6 months.^53^ The PEDI‐CAT, which is based on the PEDI, was refined through Rasch modelling, with items that extended the age range up to 20 years.^54^ It uses computerized adaptive testing to minimize the time for completion. Both the VABS and the ABAS cover a wide age range (birth to 90/89 years respectively) using parent/caregiver questionnaires. The VABS measures four domains (daily living skills, motor skills, communication, and socialization) which provide an adaptive behaviour composite (for ages from birth–6 years 11 months).^55^ The ABAS‐3 measures adaptive behaviour skills across social, practical, and conceptual domains.^56^, ^57^

The WeeFIM (child version of Functional Independence Measure) is a tool that measures the severity of disability in two main domains of motor and cognition.^58^, ^59^ It measures the type and amount of assistance required for a person with a disability to perform basic life activities effectively. It is often used to measure outcomes of rehabilitation following acquired/traumatic brain injury or burns. Training is required to be certified to use the WeeFIM.

### Neurological domain tools

Of the 15 neurological tools identified for children aged birth to 5 years, there is one screening tool and three assessment tools recommended to identify infants with a higher chance of adverse neurodevelopmental outcomes. These tools are recommended at specific timepoints for children with a history of neurological risk factors (e.g. born preterm, low birthweight, stormy neonatal period), or strong parental concern about development: (1) Prechtl's General Movements Assessment (GMA) (0–5 months of age);^60^ (2) Hammersmith Infant Neurological Examination (HINE) (3 months–2 years of age);^61^ (3) the Hand Assessment for Infants (HAI) (3–12 months of age),^62^ and (4) the Neurological, Sensory, Motor, Development Assessment (NSMDA) (>2 years of age).^63^

#### Neurological screening tools for 0 to 5 months of age

Prechtl's General Movements Assessment (GMA) is the screening tool with the best prediction of high risk of CP (98% sensitivity, specificity)^64^, ^65^, ^66^, ^67^, ^68^ and demonstrates strong ability to differentiate between infants who are typically developing and those with a higher chance of motor or cognitive delays.^69^ The GMA uses a simple 3‐ to 5‐minute video of an infant's spontaneous movements to screen infants aged from birth to 5 months corrected age.^70^ Scoring is performed by specially trained assessors. Prediction of outcomes is most accurate for GMA trajectories across time points (writhing and fidgety periods).^60^, ^69^ Emerging evidence supports the concurrent analysis using the revised version of the Motor Optimality Score^71^ to provide predictive information for risk of non‐CP adverse neurodevelopmental outcomes^72^, ^73^ and functional severity of CP.^71^, ^72^ The GMA has demonstrated feasibility both cross‐culturally and in low‐resource settings.^71^, ^74^

The Hammersmith Neonatal Neurological Examination, the Neonatal Network Neurosensory Scale, and the Amiel‐Tison Neurological Assessment at Term are not included in our recommendations as they are specifically used by specialist clinicians working in neonatal settings and have a very small time‐window for use.^66^, ^75^, ^76^, ^77^ These tools do not have evidence for use through telehealth and provide less predictive information than the GMA and HINE.

#### Neurological assessment tools for 3 months to 2 years corrected age

Infants who are screened to be at higher risk of adverse neurodevelopmental outcomes on the GMA or those who present with neurological concerns after 5 months corrected age can be referred for assessment using the HINE.^61^ The HINE can be used to assess a child up until 2 years corrected age. Compared with other neurological assessments, the HINE is inexpensive, accessible, and has excellent predictive accuracy for CP^78^ and severe motor^69^ and cognitive delay.^79^ Scores on the HINE provide further information about CP topography and prediction of gross motor skill attainment.^78^ Infants who present with five or more asymmetries on the HINE have a higher chance of unilateral CP,^80^ and warrant further investigation with a tool such as the HAI. A 2017 systematic review recommended the Test of Infant Motor Performance for early diagnosis of CP when the GMA and magnetic resonance imaging are unavailable;^15^ however, the HINE's broader age range and transdiagnostic utility for predicting outcomes other than CP support its use in preference to the Test of Infant Motor Performance.^69^, ^79^, ^81^

The HAI^62^ is recommended for early detection of unilateral CP.^82^ The HAI has strong psychometrics (Appendix S2). Accuracy of diagnosis of unilateral CP improves after 5 months of age.^80^, ^82^ Interrater and test–retest reliability are high (ICC 0.92–0.99).^83^ The HAI provides age‐normative values for unimanual and bimanual activity.^84^

#### Neurological assessment tools for >2 years

Children who present after 2 years with neurological risk factors or strong parental concerns should be referred for assessment. The recommended tool at this age is the NSMDA a combination of observation and hands‐on assessment. The NSMDA discriminates between children who are developing typically and those with mild, moderate, or severe delays predictive of neurodevelopmental outcomes.^43^ Predictive accuracy improves with increasing age.^43^

### Motor domain tools

Fourteen tools were identified and evaluated that assessed motor outcomes in children aged birth to 5 years 11 months. Eight tools evaluated fine and gross motor outcomes and six assessed gross motor development only. Recommendations include one screening tool (the Alberta Infant Motor Scale [AIMS]^85^) and four standardized assessment tools (the Peabody Developmental Motor Scales, Second Edition [PDMS‐2];^86^ Motor Assessment Battery for Children, Second Edition [MABC‐2];^87^ the Test of Gross Motor Development, Second Edition [TGMD‐2];^88^ and the Bruininks‐Oseretsky Test of Motor Proficiency [BOT‐2]^89^) to identify infants with a higher chance of ongoing motor delays.^43^

#### Motor domain 0 to 3 years corrected age

##### Motor screening < 18 months

The AIMS^85^ is an observational, norm‐referenced tool used to screen infant gross motor development until 18 months corrected age. It discriminates between infants who are developing typically and those with delays, and has good predictive accuracy at 4 months, 8 months, and 12 months to predict motor delays at 18 months.^44^ The AIMS is a reliable, cost‐effective tool which is quick and easy to administer. Owing to its observational nature, it is easily incorporated into routine clinical appointments and can be completed through telehealth.^44^ The AIMS demonstrates good concurrent validity with the motor quotients on the PDMS‐2 and the Bayley Scales of Infant Development, Second Edition at 12 months.^90^

##### Motor assessment ≤3 years

The PDMS‐2^86^ assesses a child's gross and fine‐motor skills to discriminate between those developing typically and those with mild to severe delays on the basis of a normative sample. The PDMS‐2 has very weak predictive validity at 2 years when completed before 8 months corrected age;^44^ however, at 3 years it demonstrates good predictive validity for identifying probable DCD.^91^ The PDMS‐2 has a lengthy administration time and cannot be administered through telehealth.

#### Motor domain 3 to 5 years of age

Three tools are recommended^43^ to assess motor development of children aged 3 to 5 years: the MABC‐2,^87^ the TGMD‐2,^88^ and the BOT‐2,^89^ ranked in order of predictive validity and clinical utility (Appendix S2). The MABC‐2 is a discriminative and predictive tool that assesses both fine and gross motor components and is often used for diagnostic criteria of DCD (MABC < 15th centile).^14^, ^92^ It has predictive ability and stability in the preterm population; at 4 years it predicts moderate to severe motor delay at 7 to 9 years.^91^ The MABC‐2 has a short administration time and is validated for use in telehealth up to 16 years.^93^ The MABC‐3 is now available; however, no published information of this new version was available in systematic reviews. The TGMD‐2 is a quick and feasible discriminative assessment for gross motor development in children from 3 years. It demonstrates good test–retest and interrater reliability, but lacks the ability to identify ongoing motor delays or DCD.^43^, ^91^ The TGMD‐2 has a shorter administration time (15–20 minutes) than the MABC‐2 (20–40 minutes), but does not include a fine‐motor component. The BOT‐2 is a lengthy assessment, with weaker predictive validity for later motor delay.^89^ A short form of the BOT‐2 is available which is not feasible through telehealth.

#### Fine‐motor skills assessment in the context of general motor development

Two screening tools contain fine‐motor components: the ASQ‐3 and the Denver Developmental Screening Test‐II (see the section on ‘Multi‐domain tools’). Three standardized motor assessment tools contain a fine‐motor domain (PDMS‐2, MABC‐2, BOT‐2) and three standardized multi‐domain assessment tools contain a fine‐motor domain (Bayley‐III, BDI‐2, MSEL). These are all normative tests administered in a standardized manner to discriminate whether motor skill development is typical or delayed. The fine‐motor component of the Bayley‐III before 6 months has best predictive accuracy of all the Bayley‐III domains for outcomes at 2 years of age when tracking the development of high‐risk infants born preterm.^69^ A study of concurrent validity of Bayley‐III and PDMS‐2 in children born preterm at 18 months found 97% agreement on fine‐motor skills.^94^

### Sensory domain tools

Sensory dysregulation refers to atypical responses to sensory stimuli, including inability to organize and regulate sensory stimuli (including touch, hearing, vision, smell, movement, and balance) and is often observed in children with neurodevelopmental disorders.^95^ Sensory measures are generally questionnaires completed by a parent/caregiver.

#### Sensory domain <2 years corrected age

A systematic review of sensory measures for children aged younger than 2 years recommended the Sensory Profile 2.^96^ The Sensory Profile 2 is a questionnaire for use in infants up to 6 months of age and a toddler version for 7 to 35 months of age which can be used through telehealth. The Test of Sensory Function in Infants is a performance‐based assessment that measures sensory systems (vision, tactile pressure, vestibular) for children aged 4 to 18 months of age (Appendix S2).^96^

#### Sensory domain >2 years

A recent systematic review^95^ recommended the Participation and Sensory Environment Questionnaire‐Home (PSEQ‐H) to evaluate sensory dysregulation between 2 years and 7 years.^97^ The PSEQ‐H has moderate to high quality for structural validity, hypothesis testing for construct validity, internal consistency, reliability, and measurement error. The PSEQ‐H is freely available online.^95^ Both the Child Sensory Profile‐2^95^, ^98^ and the Sensory Processing Measure ‐ Preschool were noted to have sufficient ratings of discriminant validity with moderate levels of evidence.^95^ The Child Sensory Profile‐2 was designed for children aged 3 years to 14 years 11 months to discriminate between children who had a diagnosis of autism, ADHD, or other neurodevelopmental disorders.^95^ It lacks evidence for content validity. The SPM‐P is used for 2‐ to 5‐year‐old children.

### Cognitive domain tools

Intellectual disability is characterized by deficits in general mental abilities, such as problem‐solving, planning, reasoning, and judgement.^1^ Three key criteria are used to establish a diagnosis of intellectual disability: (1) deficits in intellectual functions, confirmed by both clinical assessment and individualized, standardized intelligence testing; (2) deficits in adaptive functioning that limit functioning in one or more activities of daily life, across multiple environments; and (3) onset of intellectual and adaptive deficits during development.^1^ Global developmental delay is a diagnosis used for children younger than 5 years.^1^ Children who fail to meet expected developmental milestones in several areas of functioning, or who are unable to undergo systematic assessments of intellectual functioning, or are too young to participate in standardized testing, may receive a diagnosis of global developmental delay.^1^

A 2016 meta‐analysis of early developmental assessment tools used to assess cognition in children born very preterm or with very low birthweight found the Bayley and the revised version of the Griffiths Mental Developmental Scales were the most commonly used cognitive tests between 18 months and 40 months of age.^99^ Early assessments (both Bayley and the revised version of the Griffiths Mental Developmental Scales) were fairly accurate in predicting the absence of school‐age cognitive deficits (high negative predictive value, range 47.8–95.5%); however, prediction of any cognitive impairment was weak, with a pooled sensitivity 55% (95% CI 45.7–63.9%) and specificity 84.1% (77.5–89.1%).^99^ Meta‐analysis suggested half of children who may have cognitive difficulties at school‐age were classified as having typical neurodevelopmental function at 1 to 3 years of age.^99^ This highlights the importance of repeated assessments for children whose families/carers have concerns about their child's development.

Comprehensive measures of cognition were noted as the Wechsler Preschool and Primary Scale of Intelligence, Fourth Edition (WPPSI‐IV), the Stanford‐Binet Intelligence Scales, Fifth Edition (SB‐5), the Differential Ability Scales, Second Edition (DAS‐II), Developmental NEuroPSYchological Assessment (NEPSY‐II), and the revised version of the Leiter International Performance Scale.^22^, ^46^, ^100^, ^101^, ^102^, ^103^, ^104^ These may all be used in primary health settings by appropriately qualified professionals. The WPPSI‐IV is a measure of cognitive development for preschoolers and young children (2 years 6 months–3 years 11 months; 4 years–7 years 7 months). Validity studies of the WPPSI‐IV with other measures for targeted populations (e.g. children with ADHD, autism, intellectual disability) are published in the manual.^105^ The WPPSI‐III correlated highly with the Bayley Scales of Infant Development, Second Edition and DAS (>0.8) in studies of children with prenatal exposure to anti‐depressants.^22^ The WPPSI‐IV has been standardized for children aged 2 years 6 months to 7 years 7 months.^105^ The SB‐5 (Early SB5) comprehensively measures cognitive ability of children aged 2 years to 7 years 3 months. The Full‐scale IQ consists of 10 subtests, with a verbal and non‐verbal subtest for five factors: fluid reasoning, knowledge (including vocabulary), quantitative reasoning, visual–spatial processing, and working memory. The Stanford Binet Intelligence Scale has good test–retest and interrater reliability (*α* = 0.83).^22^ The DAS‐II measures general cognitive abilities for children aged 2 years 6 months to 17 years 11 months.^22^, ^102^ The subtests for the Early Years battery differ from that of the School‐Age battery. The Early Years battery (2 years 6 months–3 years 5 months) contains only four subtests covering verbal and non‐verbal ability, while the upper level (3 years 6 months–6 years 11 months) contains six subtests which cover verbal, non‐verbal, and spatial ability clusters. The DAS‐II allows for quick administration.^102^

The NEPSY‐II is a comprehensive measure of neuropsychological development in children aged 3 to 16 years.^22^, ^101^ It includes assessment domains of attention and executive functioning, language, memory and learning, sensorimotor, social perception, and visuospatial processing.

The revised version of the Leiter International Performance Scale is a non‐verbal assessment for cognitive abilities for children and young people aged between 2 years and 20 years.^106^ It is suitable for children who have a communication impairment but typical visual function.^103^, ^104^ It measures cognitive abilities including fluid reasoning, visuospatial ability, short‐term memory, long‐term retrieval, processing speed, and general knowledge.^103^, ^104^

Diagnosis of an intellectual disability requires assessment of adaptive functioning. Measures of adaptive functioning commonly used to inform diagnosis of intellectual developmental disorder include multi‐domain tools such as the Vineland‐3,^55^ ABAS‐3,^56^ and PEDI‐CAT.^54^ The Vineland‐3 scales measure three broad domains of adaptive functioning: communication, daily living skills, and socialization. Telehealth assessment of cognitive functions is supported.^107^

### Communication/language domain tools

There are many skills that contribute to effective communication (speech, receptive and expressive language, social communication, fluency, voice, pre‐literacy). In the preschool years, evaluation should include screening and/assessment of speech, and receptive and expressive language. Clinical practice guidelines^12^ have identified tools available to support assessment of children's communication.^12^ The tools described in this scoping review cover multiple communication domains in a single measure. Tools that assessed only a single communicative domain were not recommended owing to their limited application, despite some having strong psychometrics (e.g. the Language Use Inventory, the Peabody Picture Vocabulary Test, the Renfrew Bus Story).

#### Communication domain screening tools

The Communication and Symbolic Behavior Scales (Infant‐Toddler; CSBS) is a parent‐completed screening questionnaire of early verbal and non‐verbal communication for children aged 6 to 24 months (up to 72 months for children with significantly delayed communication).^108^ It covers a range of communication domains including functional ability to convey different communicative intents, use of gestures, speech, receptive language, and expressive vocabulary. Raw scores can be converted to standard scores and centile ranks to identify children who require further assessment. At 12 to 17 months, the CSBS screener has fair predictive validity with language evaluation on the Preschool Language Scales, Third Edition, or the MSEL at 2 to 3 years.^109^ The CSBS (Infant‐Toddler) was recommended as a screening tool for early signs of autism at 12 months owing to its high predictive value (sensitivity = 0.93, specificity = 0.89) and clinical utility.^110^ The Hearing and Talking Scale (HATS)^18^ is a screening tool for detecting communication problems in young Indigenous Australian children aged 4 to 30 months. Validity of the HATS was performed with the ASQ‐TRAK and the Expressive Vocabulary Test, Second Edition.^17^ Similarly, the Parents' Evaluation of Listening and Understanding Measure (PLUM) is a co‐designed screening tool for screening functional auditory performance of Indigenous Australian children.^16^ The PLUM can identify children at risk of hearing and listening problems.^16^

#### Standardized speech assessment tools

The Dynamic Evaluation of Motor Speech Skill is a criterion‐referenced assessment designed to differentially diagnose severe speech sound disorders in children older than 3 years, which must be administered by a speech pathologist.^111^ It has fair predictive validity for identifying childhood apraxia of speech (sensitivity = 0.9, specificity = 0.7)^112^ and strong test–retest (ICC = 0.82), and intrarater (ICC = 0.92) and interrater reliability (ICC = 0.98).^111^, ^112^ The Diagnostic Evaluation of Articulation and Phonology differentially assesses speech in children aged over 3 years (articulation, phonology, and oral motor), and includes both screening and in‐depth assessment. Although there are limited psychometrics for the Diagnostic Evaluation of Articulation and Phonology^113^ it is a widely used assessment of speech.^12^

#### Standardized language assessment tools

The Preschool Language Scales, Fifth Edition^114^ is a norm‐referenced assessment of receptive and expressive language for children from birth to 7 years 11 months. It has good predictive validity (sensitivity = 0.91, specificity = 0.78) for children at risk of language disorder.^109^ It has strong content and construct validity.^109^ A screening version (domains of language, articulation, connected speech, social/interpersonal communication skills, fluency, and voice) was designed for use by early educators.

The Clinical Evaluation of Language Fundamentals Preschool, Second Edition^115^ and Clinical Evaluation of Language Fundamentals, Fifth Edition (CELF‐5) are widely used norm‐referenced assessments of receptive and expressive language (including vocabulary, sentences, and functional communication) for children aged 3 years 6 months to 6 years 11 months, and 5 years to 21 years respectively.^12^ Psychometrics of the Clinical Evaluation of Language Fundamentals Preschool, Second Edition report poor to fair predictive validity (sensitivity = 0.42, specificity = 0.99) for identifying language disorders (using cut‐point −2SD), and fair predictive validity for ‘at risk of language disorder’ (using cut‐point −1.25SD; sensitivity = 0.64, specificity = 0.93).^109^ The CELF‐5 has good predictive validity for identifying children ‘at risk of language disorders’ (−1.5SD; sensitivity = 0.85, specificity = 0.99), but poor predictive validity for confirmed language disorder (−2SD; sensitivity = 0.57, specificity = 1.0). It has strong content and construct validity and reliability, with limited psychometrics across other domains.^109^

### Social–emotional domain

Social–emotional development in children from birth to 5 years 11 months encompasses the attainment of expected developmental milestones in social and emotional domains as well as childhood mental health. Typical socio‐emotional development in this age group includes joint attention, social interest, pretend play, developing theory of mind (understanding perspectives of other people), developing emotional regulation, and the emergence of social skills such as taking turns or sharing. Some assessments in the social–emotional domain focus on the attainment of developmental milestones while others focus on early mental health. In early childhood, mental health is understood in terms of externalizing and internalizing behaviour. Externalizing behaviours are directed outwards and may include aggression, temper tantrums, and non‐compliance. Internalizing behaviours are directed inwards and may include feelings of sadness or anxiety, social withdrawal, or physiological symptoms such as stomach aches.

There is overlap between both the developmental and the mental health aspects of the social–emotional domain and the ADHD and autism‐related symptomology which are reported under the specific diagnostic groups. Assessments are recommended on the basis of their psychometric properties and clinical utility. Some assessments have age ranges beyond 5 years 11 months of age (the Ages & Stages Questionnaires: Social–Emotional, Second Edition [ASQ‐SE‐2], and Strengths and Difficulties Questionnaire [SDQ]), so the psychometrics reported may be an underestimation, reflective of limited studies in children of this age. Assessment choice must consider the focus of the assessment: developmental attainment or mental health or both. The child's age must be taken into account, and whether it is desirable to choose an assessment that can be repeated later.

#### Social–emotional screening tools

The Brief Infant Toddler Social and Emotional Assessment (BITSEA),^116^ the SDQ, and the ASQ‐SE‐2 are recommended screening tools for social–emotional outcomes. The BITSEA is a brief screener version of the Infant Toddler Social and Emotional Assessment (ITSEA).^116^, ^117^ It is a short caregiver‐report measure for children 1 to 3 years of age. It gives total scores for social–emotional problems (including internalizing and externalizing behaviour) and social–emotional competence. A systematic review^24^ identified good to excellent internal consistency and agreement with the Child Behaviour Checklist (CBCL) (0.51–0.79), and prediction of CBCL and ITSEA scores 1 year later (Appendix S2). The ASQ‐SE‐2 is a caregiver questionnaire for children from 1 month to 6 years to assess social–emotional competency across self‐regulation, compliance, social communication, adaptive functioning, autonomy, affect, and social interaction.^118^ The ASQ‐SE‐2 has excellent internal consistency (0.84) and interrater reliability (0.91), high agreement with other measures (0.81–0.95), and good to very good predictive validity (sensitivity = 0.81, specificity = 0.84).^24^ The SDQ^119^ is a brief caregiver‐report screening tool of internalizing and externalizing behaviour as well as prosocial behaviours and peer relations for children from 2 to 4 years, and from 4 to 17 years. It is a 25‐item measure of child behaviour and adjustment. The SDQ has adequate internal reliability (*α* = 0.73) and test–retest reliability (*r* = 0.62) as well as discriminant and concurrent validity.^120^ The SDQ is freely available, with no restrictions on who can administer it.

#### Social–emotional assessment tools

Tools recommended for assessment of social–emotional outcomes in early childhood are the Behaviour Assessment System for Children (BASC), CBCL, Greenspan Social–Emotional Growth Chart, and ITSEA.^10^, ^24^, ^121^

The BASC is a widely used caregiver report for children 2 years and older.^122^ The third edition, the BASC‐3, measures externalizing and internalizing symptoms as well as social well‐being with four composite scales for preschool children: internalizing problems, externalizing problems, adaptive skills, and the behavioural symptoms index. The BASC‐2 has fair internal consistency and fair to good construct validity.^121^ The CBCL is a parent or caregiver‐report questionnaire, and the preschool version is appropriate for children aged 1 year 6 months to 5 years.^123^ It is a mental health measure, assessing both externalizing and internalizing behaviour. The CBCL is part of the Achenbach System of Empirically Based Assessment. A systematic review found the CBCL correctly classified 84% of children with emotional and behavioural problems.^24^ The Greenspan Social–Emotional Growth Chart is a caregiver‐completed questionnaire used from birth to 3 years 6 months.^124^ It is a developmental assessment, focused on the child's mastery of developmental tasks in the social–emotional domains. It has excellent internal consistency (*α* = 0.83–0.94), good construct validity as measured by agreement with the Bayley‐III, and predictive validity (sensitivity = 0.87, specificity = 0.90) as a screening tool for ASD.^24^, ^125^ The ITSEA is a caregiver‐report measure for children aged 1 to 3 years.^117^ It is both a mental health and developmental assessment covering externalizing and internalizing behaviour as well as competence across play, empathy, and prosocial peer relations. The ITSEA has good to excellent internal consistency (externalizing 0.66, internalizing 0.85, competence 0.56–0.79) and agreement with the CBCL (range 0.41–0.60).^24^

The Indigenous Child‐Initiated Pretend Play Assessment with its component of the Indigenous Play Partner Scale is an assessment of play skills and social behaviours which has been validated for Australian Aboriginal children.^18^, ^126^ Further information on this tool is given in Appendix S4.

### Tools specific to diagnostic groups

#### Cerebral palsy

CP is diagnosed on the basis of a combination of clinical and neurological signs.^4^ The International Clinical Practice Guidelines for the early, accurate diagnosis of CP found that early diagnosis begins with medical history then use of standardized neurological and motor assessments together with neuroimaging.^15^ Before 5 months corrected age, the most predictive tools are the GMA (sensitivity = 0.98)^60^ and the HINE (sensitivity = 0.90).^61^ After 5 months of age, the most predictive tools were the HINE (sensitivity = 0.90)^61^ and the Developmental Assessment of Young Children (sensitivity = 0.83).^15^, ^127^ Any infant unable to sit independently by 9 months, presenting with hand function asymmetry, or unable to take weight through the plantar surface of the feet, should receive an investigation for CP.^15^ The functional consequences of CP are described using classifications systems: the Gross Motor Function Classification System,^128^, ^129^ the Manual Ability Classification System,^130^ and the Communication Function Classification System.^131^ Each comprises five ordinal levels to discriminate meaningfully on a child's ability. Level I represents the highest level of ability and level V represents the lowest. The Gross Motor Function Measure (GMFM)^132^ is a condition‐specific measure of the capacity for gross motor activity in children aged 5 months to 16 years with a diagnosis of CP. The extensively validated versions include the original GMFM‐88 item, and the GMFM‐66 item which was developed and validated by Rasch analysis.^35^, ^58^ A recent systematic review^58^ identified that the GMFM‐66 and GMFM‐88 have sufficient responsiveness. Ceiling effects in children with high motor ability occur as the most difficult items are at a level that a typically developing 5‐year‐old can achieve. The GMFM‐66 can discriminate between children with CP who have different levels of motor ability.^133^, ^134^

#### Autism

National guidelines for screening and assessing autism are recommended for clinicians working with children at risk of autism.^13^ The guidelines highlight the importance of comprehensive multidisciplinary assessment for autism, gathering information through a combination of observation, and standardized and non‐standardized tools.

##### Screening tools for autism

There are several screening tools that can be used to identify children under the age of 5 years showing early signs of autism: the revised version of the Social Attention and Communication Surveillance (SACS‐R); the Social Attention and Communication Surveillance Preschool (SACS‐PR);^135^ the revised version of the Modified Checklist for Autism in Toddlers, with Follow‐up (M‐CHAT‐R/F);^136^ and a freely available smartphone app for parents, ASDetect.^135^, ^137^, ^138^, ^139^, ^140^, ^141^, ^142^, ^143^ The SACS‐R is recommended as the most effective screening tool from 11 to 30 months, and the SACS‐PR for children aged 3 years 6 months to 5 years.^135^ The SACS‐R is an observational checklist completed by trained clinicians which takes approximately 10 to 15 minutes to administer. The SACS‐R has strong predictive validity.^135^ The M‐CHAT‐R/F^136^ is a freely available clinician‐led parent questionnaire that can be used to screen children 16 to 30 months.^141^, ^142^, ^143^ Although the sensitivity and specificity of this tool are not as reliable as those tools above, the M‐CHAT‐R/F is readily accessible and does not require any training or costs. A meta‐analysis (*n* = 49 841) found the M‐CHAT‐R/F had a pooled sensitivity of 82.6% and specificity of 45.7%, with more effective predictive value when used in high‐risk populations.^144^ The M‐CHAT‐R/F had a sensitivity of 73% and specificity of 89% for diagnosis of autism when a cut‐off score of 3 was used with a sample of 16 071 children.^145^ Children identified as being at risk of autism using these screening tools should be referred for further diagnostic assessment and encouraged to access early intervention and family supports. ASDetect is a freely available smartphone app designed for parents and caregivers on the basis of the SACS‐R.^137^ The app provides video examples to help parents/caregivers answer questions related to their child and takes 20 to 30 minutes to administer. If a child is identified as having a high likelihood of autism, parents can seek referral from their medical practitioner for further assessment and support.

##### Diagnostic tools for autism

Many of the recommended diagnostic assessment tools require significant training and expertise. It is best practice that diagnostic assessments are done by a multidisciplinary team rather than an individual practitioner.^13^, ^146^, ^147^, ^148^, ^149^ Diagnostic tools that most accurately diagnose autism in preschool children identified in a Cochrane review^149^ were the Autism Diagnostic Observational Schedule, Second Edition (ADOS‐2),^150^ the Childhood Autism Rating Scale (CARS),^151^ and the revised version of the Autism Diagnostic Interview (ADI‐R).^152^ The ADOS is best for not missing children who have autism and is similar to the CARS and ADI‐R in not falsely diagnosing autism. Diagnostic tools should be combined with observations and information from a range of sources by specialized clinicians as recommended in guidelines.^13^

The ADOS‐2 is the recommended standardized assessment tool for autism diagnosis used with children aged 12 months and above.^147^, ^149^ This is a standardized, behavioural observation and play assessment that takes 40 to 60 minutes to administer. Meta‐analysis of the ADOS‐2 found a sensitivity of 0.94 (95% CI 0.89–0.97) and specificity of 0.80 (95% CI 0.68–0.88).^149^ Overdiagnosis is likely if the ADOS is used in populations with lower prevalences of autism.^149^ The CARS‐2 combines an interview with the parents/carers and observation of the child. It is used with children 2 years and older.^151^ The CARS‐2 has a combined sensitivity of 0.8 (95% CI 0.61–0.91) and specificity of 0.88 (95% CI 0.64–0.96).^149^ The ADI‐R is a diagnostic interview that can be used with children 2 years and older.^152^ The Autism Observation Scale for Infants^153^ is the most reliable tool for detecting autism in high‐risk populations under 36 months (e.g. children with an older sibling who has a diagnosis of autism; sensitivity = 0.84, specificity = 0.98).^145^ The Autism Observation Scale for Infants is an observational checklist completed by clinicians, which takes 15 to 20 minutes.^153^ This tool is only used in research and has not been standardized as a clinical screening or diagnostic tool.

#### ADHD

ADHD is a neurodevelopmental disorder in which persistent inattention and/or hyperactivity/impulsivity affect functioning.^1^ It begins in childhood, and features must be present before 12 years. For the inattentive presentation, at least six symptoms associated with inattention must be present such as difficulty with sustaining attention in tasks, not following through on instructions, difficulty with organizing tasks, or not paying attention to details and making careless mistakes. Diagnosis of the hyperactive/impulsive presentation requires at least five symptoms, such as fidgetiness, excessive talking, blurting out responses, restlessness, and difficulty sitting still.^1^ For all presentations, symptoms must be present across two or more settings. Symptoms must interfere with or reduce quality of functioning.^1^ Recent clinical practice guidelines on the diagnosis of ADHD^10^ highlight the importance of comprehensive assessment. Therefore, all the tools discussed are best considered as screening measures. Whereas some are multi‐domain scales that include measures of attention and behaviour (e.g. the BASC‐3, the Conners Early Childhood Assessment [Conners EC], the CBCL, and the SDQ), others focus on the executive function challenges associated with ADHD (Behavior Rating Index of Executive Function–Preschool [BRIEF‐P], Brown Attention Deficit Disorder Symptom Assessment Scale [BADDS]/Brown Executive Function/Attention Scales).

##### Screening tools for ADHD

The following tools have potential utility for screening and/or assessment for ADHD in children up to 5 years 11 months:^10^, ^154^, ^155^, ^156^ the BASC‐3,^122^ BRIEF‐P,^157^ BADDS,^158^ CBCL,^154^ Conners EC,^159^ and SDQ.^119^

The BASC‐3 is a tool used for social–emotional assessment and ADHD. It measures children's adaptive and problem behaviours and emotional difficulties at home and in community settings using parent and teacher questionnaires.^122^ Scores provide clinical and adaptive scales including attention problems and hyperactivity. Although an ADHD probability index is not calculated for 2‐ to 5‐year‐olds, an index score for clinical probability and functional impairment is provided.^122^ The BRIEF‐P measures behaviours associated with executive function in preschool children, aged 2 years to 5 years 11 months. A rating form can be completed by parents, educators, and carers to assess executive function at home and in childcare, kindergarten, or preschool settings. Domains of executive functioning, including working memory, emotional control, inhibition, shifting, and ability to plan/organize, are measured. The BRIEF‐P has high internal consistency reliability (0.80–0.95 for the parent version and 0.90–0.97 for the teacher version). Clinical utility of the BRIEF‐P to screen for ADHD in young children found thatof the five subscales, scores on the ‘Inhibit’ and ‘Working memory’ subscales were closely related to ADHD symptoms and correctly identified 86.4% of children in ADHD and typically developing groups.^160^ The BADDS and the revised version of the Brown Executive Function/Attention Scales consider problems a child who has ADHD might experience with executive function, including organization; focusing, sustaining, and shifting attention; regulating alertness; regulating emotions; working memory; and monitoring.^158^ If scores are elevated, a comprehensive clinical assessment with a clinician is appropriate. Parent and teacher versions are available for children 3 to 7 years of age.

The CBCL/1.5–5 is a broad measure, and part of the Achenbach System of Empirically Based Assessment.^161^ The CBCL/1.5–5 includes an attention syndrome scale, and a DSM‐oriented diagnostic scale for ADHD. Parent and teacher forms, with 99 items, as well as open‐ended questions gain qualitative information about a child's problems and strengths. It has been reported to have acceptable specificity (specificity = 0.81) and reasonable sensitivity (sensitivity = 0.75).^156^ The Conners EC assessment tool is used to gather parents' and teachers' observations about behaviour of children aged 2 to 6 years.^159^ It assesses behavioural, emotional, social, and developmental issues in young children. The behavioural scales include an inattention/hyperactivity scale. In addition, the Conners EC offers two global index scores that may be useful in screening for ADHD in young children: the Conners EC Global Index: Restless‐Impulsive and the Conners EC Global Index: Total. Children with elevated scores on the Conners EC Global Index: Restless‐Impulsive may be easily distracted, restless, fidgety, or impulsive. Children with elevated scores on the Conner EC Global Index: Total may be moody and emotional, restless, impulsive, and inattentive. The SDQ^120^ was summarized in screening tools in the social–emotional domain. Scores are provided for five scales: emotional symptoms, conduct problems, inattention/hyperactivity, peer problems, and prosocial behaviour. A total difficulties score is also provided. The hyperactivity/inattention subscale shows good sensitivity (sensitivity = 0.8) at the expense of specificity (specificity = 0.48).^156^ The SDQ has great utility as a brief screening assessment for attentional difficulties. The BASC‐3, CBCL, and Conners EC all provide information about attention and behaviour in the context of broader functioning and may also be useful in screening for ADHD symptoms, while also exploring general social–emotional functioning and development.

#### DCD

International clinical practice recommendations on assessment, diagnosis, and intervention of DCD^14^ recommend children under 5 years are assessed over two time points, at least 3 months apart with a strict criterion of the fifth centile cut‐off (severe impairment), and holistic consideration given to the child and their environment.^14^, ^162^ For the children scoring at or below the 16th centile on the MABC‐2, monitoring and follow‐up are recommended. The MABC‐2 is the most cited preschool‐age motor assessment for DCD^14^, ^92^, ^162^ and has best predictive validity where 4‐year‐old children with MABC‐2 scoring below the fifth centile are likely to have motor impairment at 8 years.^162^ The Developmental Coordination Disorder Questionnaire^163^ is a parent questionnaire which screens for coordination disorders in children aged 5 to 15 years; and the Little Developmental Coordination Disorder Questionnaire screens for motor coordination difficulties in 3‐ to 4‐year‐old children.^163^, ^164^, ^165^

#### FASD

FASD is a neurodevelopmental disorder associated with prenatal alcohol exposure.^11^, ^166^ The neurodevelopmental impairments affect many aspects of a child's life, including cognitive, motor, language, and social functioning.^11^ Diagnostic assessment for FASD is best conducted by a multidisciplinary team.^11^ For a diagnosis of FASD three criteria must be considered: (1) evidence of prenatal alcohol use and other exposures; (2) neurodevelopmental impairment; and (3) specific facial features.^1^, ^11^ Alternative diagnoses that might explain an individual's physical and neurodevelopmental impairments must be excluded. Forms for FASD diagnostic assessment and management planning are provided for clinical use.^11^ Multiple screening tools are summarized in a 2022 review; however, there were no specific screening tools for FASD recommended for clinical use.^166^ General developmental screening tools can be used to screen for potential neurodevelopmental impairments. Assessment should be prioritized according to the child's functional difficulties, age, and local resources.^11^ Routinely gathering information about prenatal alcohol exposure when taking a developmental history will enable accurate differential diagnosis. Collection and assessment of prenatal alcohol exposure can be undertaken using the Alcohol Use Disorders Identification Test.^167^

## DISCUSSION

This scoping review identified 67 tools with good discriminative or predictive validity for the screening and assessment of developmental concerns or disability in children from birth to 5 years 11 months of age. Early accurate identification can enable timely referral to early intervention programmes that can have a positive influence on many outcomes.^168^, ^169^, ^170^ Early accurate identification of children who have developmental concerns or a disability is the key first step to implementing successful early intervention strategies. It is well recognized that, to be effective, screening children for a disability or developmental concern should begin early and be repeated through childhood.^171^, ^172^ Screening involves the use of standardized screening tools to identify children who are at risk and may lead to more in‐depth evaluation in which history taking, standardized assessment tools, and clinical observations are used to diagnose a delay or disability. As a child's family know them best, it is vital that families are engaged as partners in the developmental screening and evaluation process.

The best screening tools recommended for use by health professionals and early educators in primary care settings include the following: for multiple developmental domains, the ASQ‐3; for autism, the SACS‐R, SACS‐PR, and ASDetect (smartphone app available for parents/caregivers); for communication, the CSBS; for social–emotional concerns, the ASQ‐SE‐2 and SDQ. These tools can be used and scored by general practitioners, maternal/child health nurses, health workers, and allied health professionals. These tools may be used in rural and remote areas through telehealth as they are parent questionnaires. The strongest evidence‐based screening tool for early detection of CP and other adverse neurodevelopmental outcomes is the GMA.

The screening tool that covers multiple developmental domains and has the best published psychometric evidence is the ASQ‐3.^28^ The ASQ‐3 can be used by all health professionals and early educators so it is well‐suited to the primary care setting. Parents and health professionals report it is acceptable and understandable.^173^ Parents report the ASQ‐3 enables them to ‘assess their own child and to work in partnership’ with health professionals.^173^ Although training is not mandatory for health professionals, it is advisable for consistency in use and scoring.^173^ Health professionals and early educators need to be aware of the limitations of the ASQ‐3 in that it is a screener, and will not identify all children with developmental concerns and may incorrectly identify others. Sensitivity and specificity of the ASQ are moderate but more accurate in older children (>24 months) than in younger ones (<24 months).^23^ As a screening tool, the ASQ items do not take into full consideration the quality of performance. For example, a young child may demonstrate asymmetry of movements that the ASQ‐3 motor domain may not discern, whereas a comprehensive motor assessment such as the HINE will more fully discern atypical motor performance. Development of clear recommendations for children who score ‘close to the cut‐off’ for two or more domains on the ASQ‐3 (e.g. communication, problem‐solving, and personal–social) would be beneficial. Intentional, systematic screening using the ASQ‐3 at the early developmental checks would benefit children from disadvantaged populations. The ASQ‐3 may be used with parents face‐to‐face, through telehealth, or online, enabling it to be utilized in urban, regional, and remote primary care settings. Use of a skills‐based checklist screener can help parents better understand their child's development.

Three multi‐domain developmental assessment tools had strong psychometrics: the Bayley‐III,^40^ the BDI‐2,^41^ and the MSEL.^42^ While the Bayley‐III is recognized as a ‘criterion standard’ in validity studies, it is limited to a ceiling age of 3 years 6 months. The MSEL can be used up to 5 years 8 months, while the BDI‐2 extends up to 7 years 11 months. Four multi‐domain functional/adaptive assessment tools that discriminate typical versus delayed development of functional performance and adaptive behaviour are recommended: the PEDI,^53^ PEDI‐CAT,^54^ VABS‐3,^55^ and ABAS‐3.^56^ These four functional assessments are parent‐report questionnaires which may be done online, or by interview through telehealth, providing greater flexibility for assessment remotely. The VABS‐3 and ABAS‐3 are commonly used in diagnosing intellectual disability. None of the multi‐domain tools require formal training in test procedure (although it is recommended for the Bayley); however, the tools are restricted to use by qualified health professionals trained in a relevant discipline (e.g. speech pathologist, occupational therapist, physiotherapist, psychologist). Routinely gathering information about prenatal alcohol exposure when taking a developmental history is important for consideration of FASD.

Training and professional support may be required to increase the capability of clinicians and the capacity of the workforce to utilize the tools. Tools recommended for comprehensive assessment of neurodevelopmental outcomes are the HINE and HAI (<2 years) and the NSMDA (>2 years). Motor assessment tools with sound psychometric evidence and strong clinical utility are the PDMS‐2 and MABC‐2. Gross and fine‐motor skill assessment are also contained in the multi‐domain assessment tools (Bayley‐III, BDI‐2, MESEL). Cognition domains are assessed within the multi‐domain tools, with the Bayley‐III being the most commonly used assessment of cognition for very young children. Cognitive tools with strong psychometrics such as the WPPSI‐IV and SB‐5 are only available for use by qualified psychologists.

Communication tools are many, and the recommendations for assessment tools in this review focused on tools that provided useful clinical information over multiple communication domains. The Preschool Language Scales, Fifth Edition and CELF‐5 are recommended for assessment of language, and the Diagnostic Evaluation of Articulation and Phonology and the Dynamic Evaluation of Motor Speech Skill for speech.

The BITSEA, SDQ, and ASQ‐SE‐2 can be used for screening of social–emotional concerns. Assessment tools such as the ITSEA, CBCL, and Greenspan Social–Emotional Chart are used for in‐depth assessment by suitably qualified health professionals. Tools for ADHD include the BASC‐3, BRIEF, and BADDS. Assessment tools for autism include the ADOS‐2, CARS‐2, and ADI‐R. Clinicians are referred to national guidelines for autism^13^ and ADHD.^11^ Tools for diagnoses of CP include the GMA and Motor Optimality Score‐Revised in early infancy, and the HINE, HAI, and GMFM with increasing age. For children with CP, classification of functional severity is described using the Gross Motor Function Classification System, the Manual Ability Classification System, and the Communication Function Classification System.

Early detection of developmental concerns using validated screening tools that are culturally appropriate for Indigenous communities is important as inappropriate tools may lead to over‐ or under‐recognition of children with developmental concerns.^17^, ^174^, ^175^ Culturally safe methods and tools are more frequently used in First Nations healthcare settings.^16^, ^174^ Four tools were identified that have been culturally validated for the Indigenous children in Australia: the ASQ‐TRAK, HATS, PLUM, and the Indigenous Child‐Initiated Pretend Play Assessment (with its component of the Indigenous Play Partner Scale; Appendix S4).

This scoping review aimed to provide recommendations on the best evidence‐based screening tools and/or developmental and diagnostic assessments to identify children with developmental concerns or a disability across broad international settings. Factors that complicate the ability to make comparisons of tools on the basis of predictive validity or discriminative validity are that statistics of test accuracy are determined by the type of measurement tool, the cut‐off points used in a study (e.g. 1 or 2SD below the mean), and the participants in a study (high‐risk group vs low‐risk group; age). The recommendations given are based on available data from this extensive scoping review which were drawn from systematic review findings. To maintain currency, it is recommended that the scoping review is updated every 5 years.

## CONCLUSIONS

This scoping review provides clinicians with recommendations for the best evidence‐based tools to assess developmental concerns or to diagnose disability on the basis of predictive or discriminative ability and clinical utility. The best multi‐domain screening tool is the ASQ‐3. The Bayley‐III and BDI‐2 are the best multi‐domain developmental assessments. Recommended diagnostic assessment tools for CP are the GMA and the HINE. The best diagnostic screening tool for autism is the SACS‐R. These tools and assessments have broad utility across multiple settings internationally.

## FUNDING INFORMATION

Australian Government Department of Health and Aged Care; the Merchant Charitable Foundation via the Children's Hospital Foundation (donation identifier: 10415). RNB is supported by an NHMRC Investigator grant, NHMRC Research Fellowship (1105038 RNB), University of Queensland Research Stimulus Grant (AB), NHMRC Early Career Fellowship (KB, APP1145212), Cerebral Palsy Alliance; PHD02619 (CL).

## CONFLICT OF INTEREST STATEMENT

The authors have stated that they had no interests that might be perceived as posing a conflict or bias.

## Supporting information


**Appendix S1:** Search Strategies for systematic reviews and grey literature.

**Appendix S2:** Excel Table ‐ Summary of Psychometric Properties and Clinical Utility.

**Appendix S3:** List of tools with abbreviations.

**Appendix S4:** Culturally appropriate tools for Indigenous Australian children.

**Appendix S5:** Systematic reviews included in review.

**Figure S1:** Flowchart.

## Data Availability

The data that support the findings of this study are openly available in supporting online information, published studies, and Figshare. https://doi.org/10.6084/m9.figshare.21708248.v1.
