# Supplementary material for: Clinical utility and psychometric properties of tools for early detection of developmental concerns and disability in young children: A scoping review
Source: Dev Med Child Neurol. 2024 Sep 16;67(3):286–306. doi: 10.1111/dmcn.16076 (PMC11794681; doi:10.1111/dmcn.16076)
Supplement: Supplementary file 1 — Appendix S1: Search Strategies for systematic reviews and grey literature. [file DMCN-67-286-s005.docx]

**Appendix 1 - Search Strategy for systematic reviews**

**MEDLINE Ovid**

(exp "Child, Preschool"/ OR exp Infant/ OR Child.tw. OR infant.tw. OR infants.tw. OR toddler*.tw. OR paediatric*.tw. OR pediatric*.tw. OR children.tw. OR childhood.tw. OR "0-5 years".tw. OR baby.tw. OR babies.tw. OR neonat*.tw. OR newborn*.tw. OR new-born*.tw. OR "pre-school child*".tw. OR perinat*.tw. OR "pre term birth".tw. OR prematur*.tw. OR ex-prem*.tw. OR "low birthweight".tw. OR "low birth weight".tw.)

AND

("Disability Evaluation"/ OR exp "Developmental Disabilities"/ OR exp "Disabled Children"/ OR disabilit*.tw.
 OR exp "Child Development"/ OR ((developmental.tw.) ADJ5 (disorder*.tw. OR impairment*.tw. OR outcome*.tw. OR delay*.tw.))

OR exp "cerebral palsy"/ OR "cerebral palsy".tw. OR "cerebral palsies".tw. OR "spastic quadriplegia".tw. OR "spastic diplegia".tw. OR "spastic hemiplegia".tw. OR "bilateral spastic*".tw. OR "unilateral spastic*".tw.

OR exp "Child Development Disorders, Pervasive"/ OR ASD.tw. OR autism.tw. OR autistic.tw.
 OR exp "Attention Deficit Disorder with Hyperactivity"/ OR adhd*.tw. OR "attention deficit disorder*".tw. OR "hyperkinetic syndrome*".tw. OR "attention deficit hyperactivity disorder*".tw. OR "minimal brain dysfunction*".tw. OR "attention span*".tw.

OR exp "Motor skills disorders"/ OR exp "Motor Skills"/ OR exp "Motor Activity"/ OR exp "Psychomotor Performance"/ OR "coordination impairment*".tw. OR "coordination disorder*".tw. OR dyspraxia.tw. OR locomotor.tw. OR locomotion.tw. OR "developmental coordination disorder*".tw. OR ((motor.tw.) ADJ5 (disorder*.tw. OR impairment*.tw. OR development.tw. OR outcome*.tw. OR delay*.tw. OR skill*))

OR exp "brain injuries"/ OR exp "cerebrovascular trauma"/ OR exp stroke/ OR "neonatal stroke*".tw. OR exp "Leukomalacia, Periventricular"/ OR "periventricular leukomalacia".tw. OR exp "Hypoxia-Ischemia, Brain"/ OR (hypoxic.tw. ADJ5 (ischemic.tw. OR ischaemic.tw. OR ischemia.tw. OR ischaemia.tw.) ADJ5 (brain.tw. OR cerebral.tw.OR encephalopath*)) OR "neonatal encephalopath*".tw. OR "perinatal asphyxia".tw. OR "brain lesion*".tw. OR "non progressive brain injur*".tw.
 OR exp "Spasms, Infantile"/
OR exp "Neonatal Abstinence Syndrome"/ OR "neonatal abstinence syndrome".tw. OR NAI.tw.
 OR exp "Movement disorders"/di OR exp "Physical Functional Performance"/ OR exp Gait/ OR exp "Mobility Limitation"/ OR "movement disorder*".tw.
 OR exp "Neurodevelopmental Disorders"/di OR ((neurodevelopmental.tw.) ADJ5 (disorder*.tw. OR impairment*.tw. OR outcome*.tw. OR delay*.tw.))
 OR exp "Fetal Alcohol Spectrum Disorders"/di OR "fetal alcohol syndrome".tw. OR FASD.tw. OR "fetal alcohol spectrum disorder".tw. OR "prenatal alcohol exposure".tw.

OR "physical disabilit*".tw. OR dyskinesia.tw. OR dystonia.tw. OR dystonic.tw. OR "activity limit*".tw. OR hyperreflexia.tw. OR hypertonia.tw. OR choreoathetosis.tw. OR ataxia.tw. OR "absent fidgety".tw. OR "abnormal fidgety".tw. OR "cramped synchron*".tw. OR "intrauterine growth restriction".tw. OR "infantile spasms".tw. OR "non accidental injur*".tw. OR "shaken baby".tw. OR "functional performance".tw. OR athetoid.tw. OR athetosis.tw.
 OR ((cognitive.tw.) ADJ5 (disorder*.tw. OR impairment*.tw. OR development.tw. OR outcome*.tw. OR delay*.tw.))

OR exp "Language Development Disorders"/ OR exp "Language Development"/ OR ((language.tw.) ADJ5 (disorder*.tw. OR impairment*.tw. OR development.tw. OR outcome*.tw. OR delay*.tw.))
 OR "sensory processing".tw.

OR exp "Social Skills"/ OR ((social.tw.) ADJ5 (disorder*.tw. OR impairment*.tw. OR development.tw. OR outcome*.tw. OR delay*.tw.))

OR exp "Communication Disorders"/ OR ((communication.tw.) ADJ5 (disorder*.tw. OR impairment*.tw. OR development.tw. OR outcome*.tw. OR delay*.tw.))

OR exp "Emotional Regulation"/ OR exp "Emotional Intelligence"/ OR ((emotional.tw.) ADJ5 (disorder*.tw. OR impairment*.tw. OR development.tw. OR outcome*.tw. OR delay*.tw.))

OR (("social emotional".tw. OR "social and emotional".tw.) ADJ5 (disorder*.tw. OR impairment*.tw. OR development.tw. OR outcome*.tw. OR delay*.tw.))
 )

AND

(exp "Neurologic Examination"/ OR exp "Neuropsychological Tests"/

OR "developmental assessment*".tw. OR "cognitive assessment*".tw. OR "physical assessment*".tw. OR "neurological assessment*".tw. OR "neurological measure*".tw. OR "neurological examination".tw. OR "neuromotor assessment*".tw. OR "neurobehavioural assessment*".tw. OR "neurobehavioral assessment*".tw. OR "neurodevelopmental assessment*".tw. OR "sensorimotor measure*".tw. OR "sensorimotor assessment*".tw.

OR diagnostic test*.tw. OR diagnostic tool*.tw. OR diagnostic measure*.tw.
OR assessment test*.tw. OR assessment tool*.tw. OR assessment measure*.tw.
OR validated test*.tw. OR validated tool*.tw. OR validated measure*.tw.
OR tests to diagnose.tw.
OR interview tool*.tw.
 OR "developmental screening questionnaire*".tw.

OR ((development*.tw. OR cognitive.tw. OR cognition.tw. OR physical.tw. OR neurological.tw. OR neurobehaviour*.tw. OR neurobehavior*.tw. OR neurodevelopment*.tw. OR sensorimotor.tw. OR sensory.tw.) ADJ3 (assessment*.tw. OR assesses.tw. OR measure.tw. OR measures.tw. OR test.tw. OR tests.tw.))

OR "hammersmith infant neurological examination".tw. OR HNNE.tw. OR Hammersmith Infant Neurological Examination.tw. OR HINE.tw. OR General Movements Assessment.tw. OR GMA.tw. OR Prechtl*.tw. OR "Motor Optimality Score".tw. OR MOS.tw. OR "Neonatal Assessment of the Preterm Infant".tw. OR NAPI.tw. OR "Neonatal Behavioral Assessment Scale".tw. OR NBAS.tw. OR "Hearing and Talking Scale".tw. OR HATS.tw. OR "Parent-evaluated Listening and Understanding Measure".tw. OR "Parent's evaluation of Listening and Understanding Measure".tw. OR PLUM.tw. OR "Ages and Stages Questionnaire*".tw. OR "Ages & Stages Questionnaire*".tw. OR ASQ-3.tw. OR "Bayley".tw. OR "Movement assessment battery for children".tw. OR MABC-2.tw. OR "Griffiths mental development scale*".tw. OR "Griffiths III".tw. OR "Rapid neurodevelopmental assessment".tw. OR RNDI.tw. OR ASQ-TRAK.tw. OR "ten questions".tw. OR "MacArthur-Bates Communicative Development Inventory.tw. OR MCI.tw. OR "Communication Symbolic Behaviour Scale.tw. OR CSBS.tw. OR "Renfrew Language Scales".tw. OR "Preschool Language Scales".tw. OR PLS.tw. OR "Clinical Evaluation of Language Fundamentals-3".tw. OR CELF.tw. OR "Verbal Motor Production Assessment for Children".tw. OR VMPAC.tw. OR "Diagnostic Evaluation of Articulation and Phonology".tw. OR DEAP.tw. OR "Goldman Fristoe Test of Articulation".tw. OR "Reynell Developmental Language Scales".tw. OR "Rossettti Infant-Toddler Language Scale".tw. OR "Intelligibility in context Scale".tw. OR ICS.tw. OR "Peabody Developmental Motor Scale*".tw. OR PDMS-2.tw. OR "Bruininks-Oseretsky test of Motor Proficiency-2".tw. OR BOT-2.tw. OR "McCarron Assessment of Neuromuscular Development".tw. OR MAND.tw. OR "Little Developmental Coordination Questionnaire".tw. OR "Little DCD-Q".tw. OR TEAF.tw. OR "Test of Infant Motor Performance".tw. OR TIMP.tw. OR "Alberta Infant Motor Scale".tw. OR AIMS.tw. OR "Developmental Assessment of Young Children".tw. OR DAYC.tw. OR "Movement Assessment of Infants".tw. OR MAI.tw. OR "Test of gross motor development".tw. OR TGMD*.tw. OR ("Ages and Stages".tw. AND "Social Emotional".tw.) OR ("Ages & Stages".tw. AND "Social Emotional".tw.) OR ASQ-SE*.tw. OR (Ages.tw. AND Stages.tw. AND "Talking about Raising Aboriginal Kids".tw.) OR ASQ-TRAK.tw. OR "Parents evaluation of developmental status".tw. OR PEDS.tw. OR "Strengths and Difficulties Questionnaire".tw. OR SDQ.tw. OR "Nipissing District Developmental Screen".tw. OR NDDS.tw. OR "Mullen Scales of Early Learning".tw. OR MSEL.tw. OR Vineland.tw. OR "VAB 2".tw. OR "VAB 3".tw. OR "Pediatric Evaluation of Disability Inventory".tw. OR PEDI.tw. OR PEDI-CAT.tw. OR "Beery-Buktenica".tw. OR "Beery VMI".tw. OR "Miller Assessment for Preschoolers".tw. OR WeeFIM.tw. OR "Wee FIM".tw. OR "Kaufman Assessment Battery for Children".tw. OR KABC*.tw. OR K-ABC*.tw. OR "Wechsler Preschool & Primary Scale of Intelligence".tw. OR "Wechsler Preschool and Primary Scale of Intelligence".tw. OR WPPSI*.tw. OR "Woodcock Johnson".tw. OR WJ-III.tw. OR WJ-IV.tw. OR "Infant-toddler Social and Emotional Assessment".tw. OR ITSEA.tw. OR "Brief ITSEA".tw. OR BITSEA.tw. OR "Child Behavior Checklist".tw. OR CBCL.tw. OR "Conners Early Childhood".tw. OR "Conners EC".tw. OR "Behavior Assessment System for Children".tw. OR BASC.tw. OR BASC-3.tw. OR BASC-4.tw OR "Modified Checklist for Autism in Toddlers".tw. OR M-CHAT*.tw. OR ("Autism Diagnostic Observation Schedule".tw. AND Toddler*.tw.) OR ADOS-T.tw. OR "Autism Observation Scale for Infants".tw. OR AOSI.tw. OR "Adaptive Behaviour Assessment System".tw. OR ABAS.tw. OR "Social Attention and Communication Surveillance".tw. OR SACS.tw. OR SACS-R.tw. OR "Childhood Autism Rating Scale".tw. OR CARS.tw. OR CARS-2.tw. OR "Autism Diagnostic Interview Revised".tw. OR ADI-R.tw. OR "Sensory Profile".tw. OR Connors.tw. OR "Hand Assessment for Infants" OR HAI.tw. OR "Mini Assisting Hand Assessment".tw. OR mini-AHA.tw. OR "Assisting Hand Assessment".tw. OR "Both Hands Assessment".tw. OR BoHA.tw. OR "Gross Motor Function Measure".tw. OR GMFM.tw. OR "Australian spasticity assessment".tw. OR "Australian spasticity scale*".tw. OR ASAS.tw. OR Brigance.tw. OR "Neuro-Sensory Motor Developmental Assessment".tw. OR "Neurological, Sensory, Motor, Developmental Assessment".tw OR NSMDA.tw. OR "Early Years Check In".tw. OR EYCI.tw.)

AND

(exp "Reproducibility of Results"/ OR exp Psychometrics/ OR exp "Sensitivity and Specificity"/ OR accuracy.tw. OR validity.tw. OR reliability.tw. OR verity.tw. OR sensitivity.tw. OR specificity.tw. OR "negative predictive value".tw. OR "positive predictive value".tw. OR psychometric*.tw.)

AND

(Child.ti. OR infant.ti. OR infants.ti. OR toddler*.ti. OR paediatric*.ti. OR pediatric*.ti. OR children.ti. OR childhood.ti. OR "0-5 years".ti. OR baby.ti. OR babies.ti. OR neonat*.ti. OR newborn*.ti. OR new-born*.ti. OR "pre-school child*".ti. OR perinat*.ti. OR "pre term birth".ti. OR prematur*.ti. OR ex-prem*.ti. OR "low birthweight".ti. OR "low birth weight".ti OR disabilit*.ti. OR ((developmental.ti.) ADJ5 (disorder*.ti. OR impairment*.ti. OR outcome*.ti. OR delay*.ti.)) OR "cerebral palsies".ti. OR "spastic quadriplegia".ti. OR "spastic diplegia".ti. OR "spastic hemiplegia".ti. OR "bilateral spastic*".ti. OR "unilateral spastic*".ti. OR ASD.ti. OR autism.ti. OR autistic.ti. OR adhd*.ti. OR "attention deficit disorder*".ti. OR "hyperkinetic syndrome*".ti. OR "attention deficit hyperactivity disorder*".ti. OR "minimal brain dysfunction*".ti. OR "attention span*".ti. OR "coordination impairment*".ti. OR "coordination disorder*".ti. OR dyspraxia.ti. OR locomotor.ti. OR locomotion.ti. OR "developmental coordination disorder*".ti. OR ((motor.ti.) ADJ5 (disorder*.ti. OR impairment*.ti. OR development.ti. OR outcome*.ti. OR delay*.ti. OR skill.ti.)) OR "neonatal stroke*".ti. OR "periventricular leukomalacia".ti. OR (hypoxic.ti. ADJ5 (ischemic.ti. OR ischaemic.ti. OR ischemia.ti. OR ischaemia.ti.) ADJ5 (brain.ti. OR cerebral.ti.OR encephalopath*)) OR "neonatal encephalopath*".ti. OR "perinatal asphyxia".ti. OR "brain lesion*".ti. OR "non progressive brain injur*".ti. OR "neonatal abstinence syndrome".ti. OR NAI.ti. OR "movement disorder*".ti. OR ((neurodevelopmental.ti.) ADJ5 (disorder*.ti. OR impairment*.ti. OR outcome*.ti. OR delay*.ti.)) OR "fetal alcohol syndrome".ti. OR FASD.ti. OR "fetal alcohol spectrum disorder".ti. OR "prenatal alcohol exposure".ti. OR "physical disabilit*".ti. OR dyskinesia.ti. OR dystonia.ti. OR dystonic.ti. OR "activity limit*".ti. OR hyperreflexia.ti. OR hypertonia.ti. OR choreoathetosis.ti. OR ataxia.ti. OR "absent fidgety".ti. OR "abnormal fidgety".ti. OR "cramped synchron*".ti. OR "intrauterine growth restriction".ti. OR "infantile spasms".ti. OR "non accidental injur*".ti. OR "shaken baby".ti. OR "functional performance".ti. OR athetoid.ti. OR athetosis.ti. OR ((cognitive.ti.) ADJ5 (disorder*.ti. OR impairment*.ti. OR development.ti. OR outcome*.ti. OR delay*.ti.)) OR ((language.ti.) ADJ5 (disorder*.ti. OR impairment*.ti. OR development.ti. OR outcome*.ti. OR delay*.ti.)) OR "sensory processing".ti. OR ((social.ti.) ADJ5 (disorder*.ti. OR impairment*.ti. OR development.ti. OR outcome*.ti. OR delay*.ti.)) OR ((communication.ti.) ADJ5 (disorder*.ti. OR impairment*.ti. OR development.ti. OR outcome*.ti. OR delay*.ti.)) OR ((emotional.ti.) ADJ5 (disorder*.ti. OR impairment*.ti. OR development.ti. OR outcome*.ti. OR delay*.ti.)) OR (("social emotional".ti. OR "social and emotional".ti.) ADJ5 (disorder*.ti. OR impairment*.ti. OR development.ti. OR outcome*.ti. OR delay*.ti.)) OR "developmental assessment*".ti. OR "cognitive assessment*".ti. OR "physical assessment*".ti. OR "neurological assessment*".ti. OR "neurological measure*".ti. OR "neurological examination".ti. OR "neuromotor assessment*".ti. OR "neurobehavioural assessment*".ti. OR "neurobehavioral assessment*".ti. OR "neurodevelopmental assessment*".ti. OR "sensorimotor measure*".ti. OR "sensorimotor assessment*".ti. OR diagnostic test*.ti. OR diagnostic tool*.ti. OR diagnostic measure*.ti. OR assessment test*.ti. OR assessment tool*.ti. OR assessment measure*.ti. OR validated test*.ti. OR validated tool*.ti. OR validated measure*.ti. OR tests to diagnose.ti. OR interview tool*.ti. OR "developmental screening questionnaire*".ti. OR ((development*.ti. OR cognitive.ti. OR cognition.ti. OR physical.ti. OR neurological.ti. OR neurobehaviour*.ti. OR neurobehavior*.ti. OR neurodevelopment*.ti. OR sensorimotor.ti. OR sensory.ti.) ADJ3 (assessment*.ti. OR assesses.ti. OR measure.ti. OR measures.ti. OR test.ti. OR tests.ti.)) OR "hammersmith infant neurological examination".ti. OR HNNE.ti. OR Hammersmith Infant Neurological Examination.ti. OR HINE.ti. OR General Movements Assessment.ti. OR GMA.ti. OR Prechtl*.ti. OR "Motor Optimality Score".ti. OR MOS.ti. OR "Neonatal Assessment of the Preterm Infant".ti. OR NAPI.ti. OR "Neonatal Behavioral Assessment Scale".ti. OR NBAS.ti. OR "Hearing and Talking Scale".ti. OR HATS.ti. OR "Parent-evaluated Listening and Understanding Measure".ti. OR "Parent's evaluation of Listening and Understanding Measure".ti. OR PLUM.ti. OR "Ages and Stages Questionnaire*".ti. OR "Ages & Stages Questionnaire*".ti. OR ASQ-3.ti. OR "Bayley".ti. OR "Movement assessment battery for children".ti. OR MABC-2.ti. OR "Griffiths mental development scales".ti. OR "Griffiths III".ti. OR "Rapid neurodevelopmental assessment".ti. OR RNDA.ti. OR ASQ-TRAK.ti. OR "ten questions".ti. OR "MacArthur-Bates Communicative Development Inventory.ti. OR MCI.ti. OR "Communication Symbolic Behaviour Scale.ti. OR CSBS.ti. OR "Renfrew Language Scales".ti. OR "Preschool Language Scales".ti. OR PLS.ti. OR "Clinical Evaluation of Language Fundamentals-3".ti. OR CELF.ti. OR "Verbal Motor Production Assessment for Children".ti. OR VMPAC.ti. OR "Diagnostic Evaluation of Articulation and Phonology".ti. OR DEAP.ti. OR "Goldman Fristoe Test of Articulation".ti. OR "Reynell Developmental Language Scales".ti. OR "Rossettti Infant-Toddler Language Scale".ti. OR "Intelligibility in context Scale".ti. OR ICS.ti. OR "Peabody Developmental Motor Scale*".ti. OR PDMS-2.ti. OR "Bruininks-Oseretsky test of Motor Proficiency-2".ti. OR BOT-2.ti. OR "McCarron Assessment of Neuromuscular Development".ti. OR MAND.ti. OR "Little Developmental Coordination Questionnaire".ti. OR "Little DCD-Q".ti. OR TEAF.ti. OR "Test of Infant Motor Performance".ti. OR TIMP.ti. OR "Alberta Infant Motor Scale".ti. OR AIMS.ti. OR "Developmental Assessment of Young Children".ti. OR DAYC.ti. OR "Movement Assessment of Infants".ti. OR MAI.ti. OR "Test of gross motor development".ti. OR TGMD*.ti. OR ("Ages and Stages".ti. AND "Social Emotional".ti.) OR ("Ages & Stages".ti. AND "Social Emotional".ti.) OR ASQ-SE*.ti. OR (Ages.ti. AND Stages.ti. AND "Talking about Raising Aboriginal Kids".ti.) OR ASQ-TRAK.ti. OR "Parents evaluation of developmental status".ti. OR PEDS.ti. OR "Strengths and Difficulties Questionnaire".ti. OR SDQ.ti. OR "Nipissing District Developmental Screen".ti. OR NDDS.ti. OR "Mullen Scales of Early Learning".ti. OR MSEL.ti. OR Vineland.ti. OR "VAB 2".ti. OR "VAB 3".ti. OR "Pediatric Evaluation of Disability Inventory".ti. OR PEDI.ti. OR PEDI-CAT.ti. OR "Beery-Buktenica".ti. OR "Beery VMI".ti. OR "Miller Assessment for Preschoolers".ti. OR WeeFIM.ti. OR "Wee FIM".ti. OR "Kaufman Assessment Battery for Children".ti. OR KABC*.ti. OR K-ABC*.ti. OR "Wechsler Preschool & Primary Scale of Intelligence".ti. OR "Wechsler Preschool and Primary Scale of Intelligence".ti. OR WPPSI*.ti. OR "Woodcock Johnson".ti. OR WJ-III.ti. OR WJ-IV.ti. OR "Infant-toddler Social and Emotional Assessment".ti. OR ITSEA.ti. OR "Brief ITSEA".ti. OR BITSEA.ti. OR "Child Behavior Checklist".ti. OR CBCL.ti. OR "Conners Early Childhood".ti. OR "Conners EC".ti. OR "Behavior Assessment System for Children".ti. OR BASC.ti. OR BASC-3.ti. OR BASC-4.ti OR "Modified Checklist for Autism in Toddlers".ti. OR M-CHAT*.ti. OR ("Autism Diagnostic Observation Schedule".ti. AND Toddler*.ti.) OR ADOS-T.ti. OR "Autism Observation Scale for Infants".ti. OR AOSI.ti. OR "Adaptive Behaviour Assessment System".ti. OR ABAS.ti. OR "Social Attention and Communication Surveillance".ti. OR SACS.ti. OR SACS-R.ti. OR "Childhood Autism Rating Scale".ti. OR CARS.ti. OR CARS-2.ti. OR "Autism Diagnostic Interview Revised".ti. OR ADI-R.ti. OR "Sensory Profile".ti. OR Connors.ti. OR "Hand Assessment for Infants" OR HAI.ti. OR "Mini Assisting Hand Assessment".ti. OR mini-AHA.ti. OR "Assisting Hand Assessment".ti. OR "Both Hands Assessment".ti. OR BoHA.ti. OR "Gross Motor Function Measure".ti. OR GMFM.ti. OR "Australian spasticity assessment".ti. OR "Australian spasticity scale*".ti. OR ASAS.ti. OR Brigance.ti. OR "Neuro-Sensory Motor Developmental Assessment".ti. OR "Neurological, Sensory, Motor, Developmental Assessment".ti OR NSMDA.ti OR "Early Years Check In".ti. OR EYCI.ti. OR accuracy.ti. OR validity.ti. OR reliability.ti. OR verity.ti. OR sensitivity.ti. OR specificity.ti. OR "negative predictive value".ti. OR "positive predictive value".ti. OR psychometric*.ti.)
 NOT (exp animals/ not humans.sh.)

NOT (exp "Diagnostic Techniques, Neurological"/ not exp "Neurologic Examination"/)

NOT (case reports.pt. OR randomized controlled trial.pt. OR clinical trial.pt. OR controlled clinical trial.pt.)
 AND

(((systematic review.ti. OR systematic literature review.ti. OR systematic scoping review.ti. OR systematic narrative review.ti. OR systematic qualitative review.ti. OR systematic evidence review.ti. OR systematic quantitative review.ti. OR systematic meta-review.ti. OR systematic critical review.ti. OR systematic mixed studies review.ti. OR systematic mapping review.ti. OR systematic cochrane review.ti. OR (systematic search AND review.ti.) OR systematic integrative review.ti.)

NOT comment.pt.

NOT (protocol.ti. OR protocols.ti.)

NOT medline.st.)

OR cochrane database of systematic reviews.jn.

OR systematic review.pt.)
Limited to English

Limited to 2000-2022

**Appendix 1 cont.**

**Search strategy for grey literature**

Grey literature search performed by one author (AB) with guidance from researcher librarian (DH).

Google searches used a phrase (e.g., “diagnosis of autism in children in Australia”). The first four pages of search results were examined for relevant information. A table was developed to record relevant information. Headings in the table were: search phrase used, relevant information/site found, what the site led to, tools/guidelines or resources. An example is shown below.

| **Search phrase** | **Found** | **Led to** | **Tools/Guidelines/Resources** |
| --- | --- | --- | --- |
| **Diagnosis of autism in children in Australia** | Royal Childrens  https://www.rch.org.au/autism/autism_assessment/Autism_assessment_ages_0-6/ | <https://www.autismcrc.com.au/access/national-guideline>  [https://www.ndis.gov.au/understanding/ families-and-carers/early-childhood-approach](https://www.ndis.gov.au/understanding/%20families-and-carers/early-childhood-approach) | National guidelines |
|  | https://www1.racgp.org.au/newsgp/clinical/australia-develops-world-s-most-effective-autism-s | the [Social Attention and Communication Surveillance-Revised (SACS-R)](https://www.latrobe.edu.au/otarc/research/autism-detection-diagnosis/social-attention-communication) | SACS-R |
|  | https://bmjopen.bmj.com/content/6/9/e012517 |  |  |
|  | https://theconversation.com/new-autism-guidelines-aim-to-improve-diagnostics-and-access-to-services-104929 | <https://www.autismcrc.com.au/access/national-guideline> |  |
|  | <https://asdetect.org/> |  | ASDetect  (app on phone) |
|  | https://www.9news.com.au/national/melbourne-scientists-create-new-screening-test-to-help-diagnose-autism-in-babies/585ee867-fef6-4998-8011-bd89e7f78095 |  | SACS |
