# Supplementary material for: Clinical utility and psychometric properties of tools for early detection of developmental concerns and disability in young children: A scoping review
Source: Dev Med Child Neurol. 2024 Sep 16;67(3):286–306. doi: 10.1111/dmcn.16076 (PMC11794681; doi:10.1111/dmcn.16076)
Supplement: Supplementary file 3 — Appendix S3: List of tools with abbreviations. [file DMCN-67-286-s006.docx]

**Appendix 3.**

Abbreviation of tool, full name of tool, included/excluded.

Black indicates tool was included/recommended and red indicates tool was excluded/not recommended.

|  | Abbreviated name | Full Name | Inc/Exc |
| --- | --- | --- | --- |
| 1 | ABAS-3* | Adaptive Behaviour Assessment System, 3rd ed | I |
| 2 | ABC | Autism Behaviour Checklist | E |
| 3 | ABILHAND-Kids | ABILHAND-Kids | E |
| 4 | ACE | Assessment of Comprehension and Expression | E |
| 5 | ACHS | Assessment of Children’s Hand Skills | E |
| 6 | ADEC | Autism Detection in Early childhood assessment | E |
| 7 | ADI-R | Autism Diagnostic interview-revised | I |
| 8 | ADOS | ADOS (T/D) | I |
| 9 | AIMS | Alberta Infant Motor Scale | I |
| 10 | ALL | Assessment of Literacy and Language | E |
| 11 | AMSE | Autism Mental Status Exam | E |
| 12 | AOSI | Autism Observation Scale for Infants | I |
| 13 | AP | Apraxia Preschool Version | E |
| 14 | APAB | Assessment of Preterm Infant Behaviour | E |
| 15 | APSI | Autism Parents Screen for Infants | E |
| 16 | AQ | Autism Questionnaire | E |
| 17 | ARI | Affective Reactivity Index | E |
| 18 | ASDetect | ASDetect - phone app parents | I |
| 19 | ASQ-3 | Ages & Stages Questionnaire | I |
| 20 | ASQ-SE | Ages & Stages Questionnaire-Social-Emotional | I |
| 21 | ASQ-TRAK^#^ | ASQ-Talking about Raising Aboriginal Kids | I |
| 22 | ATNAT | Amiel-Tison Neurological Assessment at Term | E |
| 23 | AUDIT-C* | The AUDIT Alcohol Consumption Questions (AUDIT-C) | I |
| 24 | BADDS | Brown Attention Deficit Disorder Symptom Scales | I |
| 25 | BASC-3 | Behaviour Assessment System for Children-3 | I |
| 26 | Battelle | Battelle Developmental Inventory-2 | I |
| 27 | Bayley-III | Bayley Scales of Infant and Toddler Development-III | I |
| 28 | BFMT | Baecke Fassaart Motor Test | E |
| 29 | BGMA | Basic Gross motor assessment | E |
| 30 | BIQ | Behavioural Inhibition Questionnaire | E |
| 31 | BISCUIT-Part3 | Baby and Infant Screen for Children with aUtIsm Traits—Part 3 | E |
| 32 | BITSEA | Brief Infant Toddler Social Emotional Assessment | I |
| 33 | BOT-2 | Bruininks-Oseretsky Test of Motor Proficiency -2 | I |
| 34 | BPSC | Baby Pediatric Symptom Checklist | E |
| 35 | BRIEF | Behavioural Rating Inventory of Executive Function | E |
| 36 | BRIEF-P | Behavioural Rating Inventory of Executive Function-Preschool | I |
| 37 | BRIEF-SR | Behavioural Rating Inventory of Executive Function-Self report | E |
| 38 | Brigance | Brigance Preschool Screen (not turn up in reviews but has poor validation in Aboriginal children) | E |
| 39 | BS | Behavioural Sample | E |
| 40 | CAMPB | Combined assessment of motor performance and behaviour | E |
| 41 | CARS | Childhood Autism Rating Scale | I |
| 42 | CASAT | CASAT | E |
| 43 | CASD | Checklist for Autism Spectrum Disorder | E |
| 44 | CASL | Comprehensive Assessment of Spoken Language | E |
| 45 | CAT-CLAMS | Clinical Adaptive Test/Clinical Linguistic and Auditory Scale | E |
| 46 | CBCL | Child Behavior Checklist | I |
| 47 | CBCL-AP | Child Behavior Checklist–Attention Problem | E |
| 48 | CBRS | Child Behavior Rating Scale | E |
| 49 | CBS | Child Behaviour Scale | E |
| 50 | CCC | Children's Communication Checklist | E |
| 51 | CDI | Communicative Development Inventory | E |
| 52 | CEAS | Children's Emotional Adjustment Scale | E |
| 53 | CEFS | Children’s Executive Functions Scale | E |
| 54 | CELF | Clinical Evaluation of Language Fundamentals | I |
| 55 | CESDD | Checklist of Early Signs of Developmental Disorders | E |
| 56 | CFCS | Communication Function Classification System | I |
| 57 | CHAS-P | CHAS-P and CHAS-p/t (Children Activity scales) | E |
| 58 | CHAT | CHecklist for Autism in Toddlers (M-CHAT) | E |
| 59 | CHEQ | Children's Hand-use Experience Questionnaire | E |
| 60 | CHQ | Child Health Questionnaire | E |
| 61 | CMMS-3 | Columbia Mental Maturity Scale | E |
| 62 | Conners | Conners rating Scale-Revised | I |
| 63 | CSBS ITC | Communication and symbolic Behaviour scales (Infant Toddler Checklist) | I |
| 64 | CSBS-DP-IT-Checklist | Communication and Symbolic Behavior Scales Developmental Profile Infant-Toddler Checklist | I |
| 65 | CSP2 | Child Sensory Profile 2 | I |
| 66 | CU | Complexity of Utterances | E |
| 67 | DAS-II | Differential Ability Scales | I |
| 68 | DAYC | Developmental Assessment of young Infants | I |
| 69 | DBC-ES | Developmental Behaviour Checklist - Early Screen | E |
| 70 | DCD-Q | DCD-Q, and Little DCD | I |
| 71 | DDST | Denver Developmental Screening Test | E |
| 72 | DEAP | Diagnostic Evaluation of Articulation and Phonology | I |
| 73 | DECAIT | Devereaux Early childhood assessment for Infant and Toddlers | E |
| 74 | DELV | Diagnositic Evaluation of Language Variance | E |
| 75 | DELV | Diagnostic Evaluation of Language Variance | E |
| 76 | DEMSS | Dynamic Evaluation of Motor Speech Skills | I |
| 77 | DEX-C | Dysexecutive Questionnaire for Children | E |
| 78 | DICA | Diagnostic Interview for Children and adolescents | E |
| 79 | Do-Eat | Do-Eat (ADL test) | E |
| 80 | DOTCAC | Dynamic Occupational Therapy Cognitive Assessment for Children | E |
| 81 | DTVP | Developmental Test of Visual Perception | E |
| 82 | DP-4^%^ | Developmental Profile 4 | E |
| 83 | Dubowitz | Dubowitz Neurological Assessment of the Preterm and Full-term Infant | I |
| 84 | EAS | Emotionality, Activity, Sociability Temperament Survey | E |
| 85 | ECBI | Eyberg Child Behavior Inventory | E |
| 86 | ECSA | Early Childhood Screening Assessment | E |
| 87 | EDI | Emotion Dysregulation Inventory | E |
| 88 | EPS-I | Evaluation and Programming System for Infants and Young Children | E |
| 89 | EPYFEI | Assessment of Sensory Processing and Executive Function in Childhood | E |
| 90 | Erhardt | Erhardt Developmental Prehension Test | E |
| 91 | ESAT | Early Screening of Autistic Traits Questionnaire | E |
| 92 | ESCS | Early Social Communication Scales | E |
| 93 | ESP | Early Screening Profiles | E |
| 94 | Eyetracking | Eyetracking Tools | E |
| 95 | FOCUS | Focus on the Outcomes of Communication Under Six | E |
| 96 | FSM | Functional Strength Measurement | E |
| 97 | FST | Fluharty Screening test | E |
| 98 | FTII | Fagan Test of Infant Intelligence | E |
| 99 | FYI | First Years Inventory | E |
| 100 | GAPS | Grammar and Phonology Screening test | E |
| 101 | GAPS | Grammatical Additionally, Phonology Screening | E |
| 102 | Gesell | Gesell Developmental Schedules | E |
| 103 | GFTA | Goldman Fristoe Test of Articulation | E |
| 104 | GLS | General Language Screen | E |
| 105 | GMA | Prechtl General Movements Assessment | I |
| 106 | GMFCS | Gross Motor Function Classification System | I |
| 107 | GMFM | Gross Motor Function Measure | I |
| 108 | GMPM | Gross Motor Performance Measure | E |
| 109 | GRTR | Get Ready to Read! Screening Tool (GRTR) | E |
| 110 | GSEGC | Greenspan Social Emotional Growth Chart | I |
| 111 | GSMD | Griffith Scales of Mental Development | I |
| 112 | HAI | Hand Assessment for Infants | I |
| 113 | HATS*^#^ | Hearing and Talking Scale | I |
| 114 | HELST | Hackney Early Language Screening Test | E |
| 115 | HINE | Hammersmith Infant Neurologic Examination | I |
| 116 | HINT | Harris Infant Neuromotor Test | E |
| 117 | HNNE | Hammersmith Neonatal Neurological Examination/Dubowitz | E |
| 118 | HSQ-PDD | Home Situations Questionnaire | E |
| 119 | IBQ | Infant Behavior Questionnaire-revised | E |
| 120 | I-ChIPPA *^#^ and I-PPS | Indigenous Child-Initiated Pretend Play Assessment (and its component Indigenous Play Partner Scale) | I |
| 121 | ICS | Intelligibility in Context Scale | E |
| 122 | INIB | Infant Neurological International Battery | E |
| 123 | IPDS | Infant Psychological Development Scale | E |
| 124 | ITC | Infant Toddler Checklist | I |
| 125 | ITPA | Illinois Test of Psycholinguistic Abilities | E |
| 126 | ITSEA | Infant Toddler Social Emotional Assessment | I |
| 127 | ITSP | Infant Toddler Sensory Profile | I |
| 128 | JA-OBS | joint attention-observation schedule | E |
| 129 | KIDI | Knowledge of Infant Development Inventory | E |
| 130 | KPTC | Kaufman Praxis Test for Children | E |
| 131 | KSHQ | Knickerbocker Sensorimotor History Questionnaire | E |
| 132 | LCT | Listening Comprehension Test | E |
| 133 | LDS | Language Development Survey | E |
| 134 | Leiter-R | Leiter International Performance Scale-revised | I |
| 135 | LMLST | Levett Muir Language Screening tEst | E |
| 136 | LPS | Litre International Performance scale-revised | E |
| 137 | LUI | Language Use Inventory | E |
| 138 | MA | Melbourne Assessment | E |
| 139 | MABC-2 | Movement Assessment Battery for Children-2/ & Checklist | I |
| 140 | MACS | Manual Ability Classification System | I |
| 141 | MAI | Motor Assessment of Infancy | E |
| 142 | MAND | McCarron Assessment Neuromuscular Development | E |
| 143 | MAP | Miller Assessment for Preschoolers | E |
| 144 | MBCDI | MacArthur Bates Communication Development Inventories | E |
| 145 | McCarthy | McCarthy's scales of children's abilities | E |
| 146 | M-CHAT-R/F | The Modified Checklist for Autism in Toddlers—Revised with Follow-up | I |
| 147 | M-FUN ^%^ | Miller Function and Participation Scales | E |
| 148 | MLU | Mean Length of Utterance | E |
| 149 | mMCQ | Modified MacArthur Communication Questionnaire | E |
| 150 | MMFC | Mayes Motor-Free Compilation | E |
| 151 | MOQT | Motor Observation Questionnaire for Teachers | E |
| 152 | MOS-R | The Motor Optimality Score for 3-5 month old infants - revised (MOS-Revised) | I |
| 153 | MOT4-6 | Motor Test for Children Aged 4–6 years | E |
| 154 | MPRSD-SE | Merrill Palmer Revised Scales of Development, Social-Emotional | E |
| 155 | MSAP | Madison Speech Assessment Protocol | E |
| 156 | MSEL | Mullen Scales of Early Learning | I |
| 157 | MVPT | Motor-free visual Perceptual test | E |
| 158 | NAPI | Neurobehavioural Assessment of the Preterm Infant | E |
| 159 | NBAS | Neonatal Behavioural Assessment Scale | E |
| 160 | NCATS | Nursing Child Assessment Teaching Scale | E |
| 161 | NCBRF | Nisonger Child Behaviour Rating Form | E |
| 162 | NEPSY | NEPSY | I |
| 163 | NMBA | Neuromotor Behavioural Assessment | E |
| 164 | NNNS | Neonatal Network Neurobehavioral Scale | E |
| 165 | NODA | NODA | E |
| 166 | NPA | Neurodevelopmental Physiotherapy Assessment | E |
| 167 | NRDSL | New Reynell Developmental Scales of Language | E |
| 168 | NSMDA | Neurological Sensory Motor Developmental Assessment | I |
| 169 | NWR | Nonword repetition | E |
| 170 | OFT | Orofacial Praxis Test | E |
| 171 | OWLS | Oral and Written Language Scales | E |
| 172 | PAFMI | Posture and Fine motor assessment of Infants | E |
| 173 | PARCA-R* | Parent Report of Children's Abilities-Revised | I |
| 174 | PAS-R | Preschool Anxiety Scale Revised | E |
| 175 | PAT | Photo Articulation Test | E |
| 176 | PBQ | Preschool behavior Questionnaire | E |
| 177 | PDD-MRS | The scale of Pervasive Developmental Disorder in Mentally Retarded Persons | E |
| 178 | PDMS-2 | Peabody Developmental Motor Scales-2 | I |
| 179 | PEDI | Pediatric Evaluation of Disability Inventory | I |
| 180 | PEDI-CAT | Pediatric Evaluation of Disability Inventory-Computer Adaptive Test | I |
| 181 | PEDS | Parents’ Evaluation of Developmental Status | I |
| 182 | PIECES | Play in Early Childhood Evaluation System | E |
| 183 | PLAY | Play Based Assessment | E |
| 184 | PLS-5 | Preschool Language Scales-5 | I |
| 185 | PLUM* ^#^ | Parents evaluation of Listening and Understanding Measure | I |
| 186 | PMAL-R | Pediatric Motor Activity Log -revised | E |
| 187 | POEMS | Parent Observation of Early Markers Scale | E |
| 188 | PPVT | Peabody Picture Vocabulary test | I |
| 189 | PQ | Parental Questionnaire | E |
| 190 | PSEQ-C | Participation and Sensory Environment Questionnaire- Community | E |
| 191 | PSEQ-H | Participation and Sensory Environment Questionnaire- Home | I |
| 192 | PSOM | Pediatric Stroke Outcome Measure | E |
| 193 | PTI | Pictorial Test of Intelligence | E |
| 194 | Q-CHAT | Quantitative -Checklist for Autism in toddler | E |
| 195 | QUEST | Quality of Upper Extremity Skills Test | E |
| 196 | Range | Range | E |
| 197 | RBMT-C | Rivermead Behavioural Memory Test for children | E |
| 198 | RCPM | Raven’s Coloured Progressive Matrices | E |
| 199 | Renfrew | Renfrew Bus Story | E |
| 200 | Reynell | Reynell developmental language | E |
| 201 | SACS | Social attention and communication survey-revised /Asdetect app | I |
| 202 | SB-5 | Stanford-Binet Intelligence Scale-5 | I |
| 203 | SBQ | Sensory Behavior Questionnaire | E |
| 204 | SCQ | Social Communication Questionnaire | E |
| 205 | SDQ | Strengths & Difficulties Questionnaire | I |
| 206 | SEAEM | Social emotional assessment evaluation measure | E |
| 207 | SEQ-3 | Sensory Experiences Questionnaire- Version 3 | E |
| 208 | SIOLD | Screening for Identification of Oral Language Difficulties by Preschool Teachers | E |
| 209 | SIPT | Sensory Integration and Praxis Test | E |
| 210 | SKOLD | Screening Kit of Language Development | E |
| 211 | SLPESA | Speech and Language Pathology Early Screening Assessment | E |
| 212 | SP2 | Sensory Profile/short/Infant/short | I |
| 213 | SPELT | Structured Photographic Expressive Language Test | E |
| 214 | SPM/SPM-P | Sensory Processing Measure/ SP Measure- Preschool | I |
| 215 | SPSRC | Sensory Processing and Self- Regulation Checklist | E |
| 216 | SRS | SRS | E |
| 217 | SRS | Sensory Rating Scale | E |
| 218 | SRS | Social responsiveness scale | E |
| 219 | SRST | Sentence Repetition Screening Test | E |
| 220 | SSLM | Sure Start Language Measure | E |
| 221 | STA | Screening tool by ASHA | E |
| 222 | STDAS | Screening Test for Developmental Apraxia of Speech: 2nd edition | E |
| 223 | Stroop | Stroop Color and Word Test | E |
| 224 | TEACh-5 | TEACh-5 | E |
| 225 | TELD | Test of Early Language Development/Test of Language Development | E |
| 226 | TGMD-2 | Test of Gross Motor Development-2,3 | I |
| 227 | TIMP | Test of Infant Motor Performance | E |
| 228 | TINE | Toddler Infant Neuromotor Examination | E |
| 229 | Towen | Towen Infant Neurologic Examination | E |
| 230 | TPBA-2 | Transdisciplinary Play-Based Assessment, 2nd edition | E |
| 231 | TPT | Toddler Phonology Test | E |
| 232 | TSFI | Test of Sensory Function in Infants | I |
| 233 | TVPS-R | Test of Visual Perceptual skills revised | E |
| 234 | VABS | Vinelands Adaptive Behaviour Scale | I |
| 235 | VAS | Vanderbilt Attention Scales | E |
| 236 | VDP | Verbal Dyspraxic Profile | E |
| 237 | VMPAC | Verbal Motor Production Assessment for Children | E |
| 238 | WCST | Wisconsin Card sorting test | E |
| 239 | WeeFIM | Functional Independence Measure for Children | I |
| 240 | WISC | Wechsler Intelligence Scale for Children | E |
| 241 | Wisconsin | Wisconsin Fine Motor Steadiness Battery | E |
| 242 | Woodcock | Woodcock-Johnson Oral Language | E |
| 243 | WPPSI-II | Wechsler Preschool and Primary Scale of Intelligence | I |
| 244 | YACHT | YACHT-18 young autism and other developmental disorders checkup tool | E |
| 245 | Yale | Yale Screener | E |
| 246 | ZNA | Zurich Neuromotor Assessment | E |

*not identified in systematic review search, but included following (i) feedback from clinician/researchers and (ii) tool found to have adequate psychometric evidence

# culturally validated for Aboriginal and Torres Strait Islander population

^%^ not identified in systematic review search, was raised by clinician/researchers and found to have inadequate psychometric evidence published
