# Supplementary material for: Clinical utility and psychometric properties of tools for early detection of developmental concerns and disability in young children: A scoping review
Source: Dev Med Child Neurol. 2024 Sep 16;67(3):286–306. doi: 10.1111/dmcn.16076 (PMC11794681; doi:10.1111/dmcn.16076)
Supplement: Supplementary file 4 — Appendix S4: Culturally appropriate tools for Indigenous Australian children. [file DMCN-67-286-s003.docx]

**Appendix 4 -** **Culturally appropriate tools for Indigenous Australian children**

For clinicians in Australia, further information is provided here on tools which were culturally appropriate for Indigenous Australian children. Four tools were found which had cultural validity: The Ages & Stages Questionnaire – Talking About Raising Aboriginal Kids (ASQ-TRAK), The Hearing and Talking Scale (HATS), The Parents’ Evaluation of Listening and Understanding Measure (PLUM), and The Indigenous Child-Initiated Pretend Play Assessment (I-ChIPPA) with its component of the Indigenous Play Partner Scale (I-PPS).

Cultural validation of the ASQ for Australian Aboriginal and Torres Strait Islander children led to the development of a separate screening tool, the ASQ-TRAK (Talking About Raising Aboriginal Kids).^31,173,16^ The ASQ-TRAK uses a face-to-face caregiver interview format, with opportunity for the child to demonstrate skills. Training is required to use the ASQ-TRAK in a culturally appropriate manner. The ASQ-TRAK demonstrated acceptable concurrent validity with the Bayley Scales of Infant and Toddler Development 3^rd^ Edition (Bayley-III) and the Battelle Developmental Inventory 2^nd^ Edition (BDI-2) with sensitivity of 83%, specificity 83% and negative predictive value 99% for detecting developmental concerns in Indigenous children.^16^ The high negative predictive value means that few children with developmental difficulties are missed.^16^ The ASQ-TRAK can be used confidently to accurately detect children who are likely to have developmental difficulties. The ASQ-TRAK is being extended to include all age intervals included by the ASQ-3, however it is not yet available (when available it will be called the ASQ-TRAK2 – source ASQ-TRAK Newsletter October 2022).

The Hearing and Talking Scale (HATS)^18^ is a screening tool for detecting communication problems in young Indigenous Australian children aged 4-to-30-months. The questionnaire is appropriate for use with parents/carers of young children by community workers not trained in speech-language pathology. Validity of the HATS was performed with the ASQ-TRAK and the Expressive Vocabulary Test-2^nd^ Edition (EVT-2).^18^ Similarly, the Parents’ Evaluation of Listening and Understanding Measure (PLUM) is a co-designed screening tool for use by primary health workers and teachers to screen functional auditory performance of Indigenous Australian children.^17^ The PLUM can be used to identify children who may be at risk of hearing and listening problems.^17^ The HATS and PLUM were developed using a co-design approach, with validation studies comprised of indigenous children form urban, regional and remote communities. The HATS and PLUM are used to screen and triage young children for early referral for hearing assessments (PLUM) or for specialist communication/language assessment and intervention (HATS). Early identification of children at risk of hearing difficulties and /or communication concerns can prevent vulnerable children from suffering more severe hearing loss and address any speech and language concerns as early as possible.^17,18^ The HATS and PLUM were designed to be used in person or via telehealth. Although validated on children indigenous to Australia, these tools are highlighted as they may be of interest to other indigenous communities around the world. The HATS and PLUM were designed to be used in person or via telehealth.

The I-ChIPPA assesses imaginative and symbolic play, and Indigenous Play Partner Scale (I-PPS) examines social interactions and social behaviours during pretend play with another child.^126,19^ The Indigenous Child-Initiated Pretend Play Assessment (I-ChIPPA) with its component of the Indigenous Play Partner Scale (I-PPS) is an assessment of play skills and social behaviours which has been validated for Aboriginal children in the Pilbara region of Western Australia aged 4-to-7 years 11 months.^126,19^ The I-ChIPPA assesses imaginative and symbolic play, and I-PPS examines social interactions and social behaviours during pretend play with another child.^126,19^ Further studies are required to establish cultural and content validity of this tool.

More tools that are validated or specifically designed for Indigenous Australians are needed. The ASQ-STEPS is a new standardized measure designed to assess Aboriginal and Torres Strait Islander children’s developmental progress and is currently in the validation stage of research (Source: ASQ-TRAK Newsletter October 2022). Validation is also underway on the Rapid Neurodevelopmental Assessment (RNDA).^182^ The RNDA is a screening tool which has good face validity and high interrater reliability,^183^ however was not reported in any systematic reviews. Validation and reliability for the RNDA are based upon populations in Bangladesh and Guatemala.^184^ Based on these limitations, the RNDA was not recommended in this scoping review.
