# Supplementary material for: Clinical utility and psychometric properties of tools for early detection of developmental concerns and disability in young children: A scoping review
Source: Dev Med Child Neurol. 2024 Sep 16;67(3):286–306. doi: 10.1111/dmcn.16076 (PMC11794681; doi:10.1111/dmcn.16076)
Supplement: Supplementary file 5 — Appendix S5: Systematic reviews included in review. [file DMCN-67-286-s004.docx]

**Appendix 5 – Systematic reviews included in this scoping review**

1 Aishworiya R, Van Kim M, Stewart S, Hagerman R, Feldman HM. Meta-analysis of the Modified Checklist for Autism in Toddlers, Revised/Follow-up for Screening. *Pediatrics* 2023; **151**.

2 Albuquerque PLd, Lemos A, Guerra MQdF, Eickmann SH. Accuracy of the Alberta Infant Motor Scale (AIMS) to detect developmental delay of gross motor skills in preterm infants: A systematic review. *Developmental Neurorehabilitation* 2015; **18**: 15-21.

3 Alotaibi M, Long T, Kennedy E, Bavishi S. The efficacy of GMFM-88 and GMFM-66 to detect changes in gross motor function in children with cerebral palsy (CP): a literature review. *Disability & Rehabilitation* 2014; **36**: 617-27.

4 Bevan SL, Liu J, Wallis KE, Pinto-Martin JA. Screening Instruments for Developmental and Behavioral Concerns in Pediatric Hispanic Populations in the United States: A Systematic Literature Review. *Journal of Developmental & Behavioral Pediatrics* 2020; **41**: 71-80.

5 Bieber E, Smits-Engelsman BC, Sgandurra G, Cioni G, Feys H, Guzzetta A, Klingels K. Manual function outcome measures in children with developmental coordination disorder (DCD): Systematic review. *Research in Developmental Disabilabilities* 2016; **55**: 114-31.

6 Bosanquet M, Copeland L, Ware R, Boyd R. A systematic review of tests to predict cerebral palsy in young children. *Developmental Medicine & Child Neurology* 2013; **55**: 418-26.

7 Breidbord J, Croudace TJ. Reliability Generalization for Childhood Autism Rating Scale. *Journal of Autism & Developmental Disorders* 2013; **43**: 2855-65.

8 Burger M, Louw QA. The predictive validity of general movements - A systematic review. *European Journal of Paediatric Neurology* 2009; **13**: 408-20.

9 Burgess A, Boyd RN, Ziviani J, Sakzewski L. A systematic review of upper limb activity measures for 5‐ to 18‐year‐old children with bilateral cerebral palsy. *Australian Occupational Therapy Journal* 2019; **66**.

10 Caesar R, Colditz PB, Cioni G, Boyd RN. Clinical tools used in young infants born very preterm to predict motor and cognitive delay (not cerebral palsy): a systematic review. *Developmental Medicine & Child Neurology* 2021; **63**: 387-95.

11 Cairney DG, Kazmi A, Delahunty L, Marryat L, Wood R. The predictive value of universal preschool developmental assessment in identifying children with later educational difficulties: A systematic review. *PLoS ONE [Electronic Resource]* 2021; **16**.

12 Chang LY, Wang MY, Tsai PS. Diagnostic accuracy of Rating Scales for attention-deficit/hyperactivity disorder: A meta-analysis. *Pediatrics* 2016; **137**.

13 Chevignard MP, Soo C, Galvin J, Catroppa C, Eren S. Ecological assessment of cognitive functions in children with acquired brain injury: a systematic review. *Brain Injury* 2012; **26**: 1033-57.

14 Craciunoiu O, Holsti L. A Systematic Review of the Predictive Validity of Neurobehavioral Assessments During the Preterm Period. *Physical & Occupational Therapy in Pediatrics* 2017; **37**: 292-307.

15 Cunha ACB, Berkovits MD, Albuquerque KA. Developmental Assessment With Young Children: A Systematic Review of Battelle Studies. *Infants & Young Children: An Interdisciplinary Journal of Early Childhood Intervention* 2018; **31**: 69-90.

16 da Silva MA, de Mendonça Filho EJ, Mônego BG, Bandeira DR. Instruments for multidimensional assessment of child development: A systematic review. *Early Child Development and Care* 2020; **190**: 1257-71.

17 Darsaklis V, Snider LM, Majnemer A, Mazer B. Predictive validity of Prechtl's Method on the Qualitative Assessment of General Movements: a systematic review of the evidence. *Developmental Medicine & Child Neurology* 2011; **53**: 896-906.

18 De Roubaix A, Van de Velde D, Roeyers H, Van Waelvelde H. Standardized motor assessments before the age of five predicting school-aged motor outcome including DCD: A systematic review. *European Journal of Paediatric Neurology* 2021; **30**: 29-57.

19 Debuse D, Brace H. Outcome measures of activity for children with cerebral palsy: a systematic review. *Pediatric Physical Therapy* 2011; **23**: 221-31.

20 Desideri L, Pérez-Fuster P, Herrera G. Information and Communication Technologies to Support Early Screening of Autism Spectrum Disorder: A Systematic Review. *Children* 2021; **8**: 1-29.

21 Downs SJ, Boddy LM, McGrane B, Rudd JR, Melville CA, Foweather L. Motor competence assessments for children with intellectual disabilities and/or autism: a systematic review. *BMJ Open Sport & Exercise Medicine* 2020; **6**: e000902.

22 Duffield TC, Parsons TD, Landry A, Karam S, Otero T, Mastel S, Hall TA. Virtual environments as an assessment modality with pediatric ASD populations: a brief report. *Child Neuropsychology* 2018; **24**: 1129-36.

23 Eeles AL, Spittle AJ, Anderson PJ, Brown N, Lee KJ, Boyd RN, Doyle LW. Assessments of sensory processing in infants: a systematic review. *Developmental Medicine & Child Neurology* 2013; **55**: 314-26.

24 Elvrum AKG, Sæther R, Riphagen II, Vik T. Outcome measures evaluating hand function in children with bilateral cerebral palsy: a systematic review. *Developmental Medicine & Child Neurology* 2016; **58**: 662-71.

25 Engelmann KA, Jordan LC. Outcome measures used in pediatric stroke studies: a systematic review. *Archives of Neurology* 2012; **69**: 23-7.

26 Ferre-Fernandez M, Murcia-Gonzalez MA, Espinosa MDB, Rios-Diaz J. Measures of Motor and Functional Skills for Children With Cerebral Palsy: A Systematic Review. *Pediatric Physical Therapy* 2020; **32**: 12-25.

27 Geng X, Kang X, Wong PCM. Autism spectrum disorder risk prediction: A systematic review of behavioral and neural investigations. *Progress in Molecular Biology and Translational Science* 2020; **173**: 91-137.

28 Gerber CN, Labruyère R, van Hedel HJA. Reliability and Responsiveness of Upper Limb Motor Assessments for Children With Central Neuromotor Disorders: A Systematic Review. *Neurorehabilitation & Neural Repair* 2016; **30**: 19-39.

29 Goo M, Tucker K, Johnston LM. Muscle tone assessments for children aged 0 to 12 years: a systematic review. *Developmental Medicine & Child Neurology* 2018; **60**: 660-71.

30 Greaves S, Imms C, Dodd K, Krumlinde-Sundholm L. Assessing bimanual performance in young children with hemiplegic cerebral palsy: a systematic review. *Developmental Medicine & Child Neurology* 2010; **52**: 413-21.

31 Gridley N, Blower S, Dunn A, Bywater T, Bryant M. Psychometric Properties of Child (0-5 Years) Outcome Measures as used in Randomized Controlled Trials of Parent Programs: A Systematic Review. *Clinical Child & Family Psychology Review* 2019; **22**: 388-405.

32 Griffiths A, Toovey R, Morgan PE, Spittle AJ. Psychometric properties of gross motor assessment tools for children: A systematic review. *BMJ Open* 2018; **8**.

33 Grubb M, Golden A, Withers A, Vellone D, Young A, McLachlan K. Screening approaches for identifying fetal alcohol spectrum disorder in children, adolescents, and adults: A systematic review. *Alcoholism: Clinical & Experimental Research* 2021; **45**: 1527-47.

34 Gubiani MB, Pagliarin KC, Keske-Soares M. Tools for the assessment of childhood apraxia of speech. *CoDAS (São Paulo)* 2015; **27**: 610–5.

35 Hanratty J, Livingstone N, Robalino S, Terwee CB, Glod M, Oono IP, Rodgers J, Macdonald G, McConachie H. Systematic review of the measurement properties of tools used to measure behaviour problems in young children with autism. *PLoS ONE [Electronic Resource]* 2015; **10**.

36 Hirota T, So R, Kim YS, Leventhal B, Epstein RA. A systematic review of screening tools in non-young children and adults for autism spectrum disorder. *Research in Developmental Disabilities* 2018; **80**: 1-12.

37 Hjorth S, Bromley R, Ystrom E, Lupattelli A, Spigset O, Nordeng H. Use and validity of child neurodevelopment outcome measures in studies on prenatal exposure to psychotropic and analgesic medications - A systematic review. *PLoS ONE [Electronic Resource]* 2019; **14**.

38 Homack S, Riccio CA. A meta-analysis of the sensitivity and specificity of the Stroop Color and Word Test with children. *Archives of Clinical Neuropsychology* 2004; **19**: 725-43.

39 Hulteen RM, Barnett LM, True L, Lander NJ, del Pozo Cruz B, Lonsdale C. Validity and reliability evidence for motor competence assessments in children and adolescents: A systematic review. *Journal of Sports Sciences* 2020; **38**: 1717-98.

40 Jorquera-Cabrera S, Romero-Ayuso D, Rodriguez-Gil G, Trivino-Juarez JM. Assessment of Sensory Processing Characteristics in Children between 3 and 11 Years Old: A Systematic Review. *Frontiers in Pediatrics* 2017; **5**: 57.

41 Klingels K, Jaspers E, Van de Winckel A, De Cock P, Molenaers G, Feys H. A systematic review of arm activity measures for children with hemiplegic cerebral palsy. *Clinical Rehabilitation* 2010; **24**: 887-900.

42 Kwong AKL, Fitzgerald TL, Doyle LW, Cheong JLY, Spittle AJ. Predictive validity of spontaneous early infant movement for later cerebral palsy: a systematic review. *Developmental Medicine & Child Neurology* 2018; **60**: 480-9.

43 Lebersfeld JB, Swanson M, Clesi CD, O'Kelley SE. Systematic Review and Meta-Analysis of the Clinical Utility of the ADOS-2 and the ADI-R in Diagnosing Autism Spectrum Disorders in Children. *Journal of Autism & Developmental Disorders* 2021; **51**: 4101-14.

44 Lim YH, Watkins RE, Jones H, Kippin NR, Finlay-Jones A. Fetal alcohol spectrum disorders screening tools: A systematic review. *Research in Developmental Disabilities* 2022; **122**: 104168.

45 Mawle E, Griffiths P. Screening for autism in pre-school children in primary care: systematic review of English Language tools. *International Journal of Nursing Studies* 2006; **43**: 623-36.

46 Mendonça B, Sargent B, Fetters L. Cross-cultural validity of standardized motor development screening and assessment tools: a systematic review. *Developmental Medicine & Child Neurology* 2016; **58**: 1213-22.

47 Mensch SM, Rameckers EAA, Echteld MA, Evenhuis HM. Instruments for the evaluation of motor abilities for children with severe multiple disabilities: A systematic review of the literature. *Research in Developmental Disabilities* 2015; **47**: 185-98.

48 Moon SJ, Hwang JS, Shin AL, Kim JY, Bae SM, Sheehy‐Knight J, Kim JW, Sheehy-Knight J. Accuracy of the Childhood Autism Rating Scale: a systematic review and meta-analysis. *Developmental Medicine & Child Neurology* 2019; **61**: 1030-8.

49 Morgan C, Honan I, Allsop A, Novak I, Badawi N. Psychometric Properties of Assessments of Cognition in Infants With Cerebral Palsy or Motor Impairment: A Systematic Review. *Journal of Pediatric Psychology* 2019; **44**: 238-52.

50 Mulraney M, Arrondo G, Musullulu H, Iturmendi-Sabater I, Cortese S, Westwood SJ, Donno F, Banaschewski T, Simonoff E, Zuddas A, Döpfner M, Hinshaw SP, Coghill D. Systematic Review and Meta-analysis: Screening Tools for Attention-Deficit/Hyperactivity Disorder in Children and Adolescents. *Journal of the American Academy of Child and Adolescent Psychiatry* 2022; **61**: 982-96.

51 Muthusamy S, Wagh D, Tan J, Bulsara M, Rao S. Utility of the Ages and Stages Questionnaire to Identify Developmental Delay in Children Aged 12 to 60 Months: A Systematic Review and Meta-analysis. *Journal of American Medical Association Pediatrics* 2022.

52 Nicholls A, Williams JM. Can the Prechtl method for the qualitative assessment of general movements be used to predict neurodevelopmental outcome, at eighteen months to three years, of infants born preterm? *Physical Therapy Reviews* 2016; **21**: 131-7.

53 Noble Y, Boyd R. Neonatal assessments for the preterm infant up to 4 months corrected age: a systematic review. *Developmental Medicine & Child Neurology* 2012; **54**: 129-39.

54 O'Grady MG, Dusing SC. Reliability and Validity of Play-Based Assessments of Motor and Cognitive Skills for Infants and Young Children: A Systematic Review. *Physical Therapy* 2015; **95**: 25-38.

55 Peinado-Gorlat P, Gomez de Valcarcel-Sabater M, Gorlat-Sanchez B. General movement assessment as a tool for determining the prognosis in infantile cerebral palsy in preterm infants: a systematic review. *Revue Neurologique* 2020; **71**: 134-42.

56 Pereira A, Lopes S, Magalhães P, Sampaio A, Chaleta E, Rosário P. How executive functions are evaluated in children and adolescents with cerebral palsy? A systematic review. *Frontiers in Psychology* 2018; **9**.

57 Petrocchi S, Levante A, Lecciso F. Systematic review of level 1 and level 2 screening tools for autism spectrum disorders in toddlers. *Brain Sciences* 2020; **10**.

58 Pires CDS, Marba STM, Caldas JPDS, Stopiglia MDCS. Predictive value of the general movements assessment in preterm infants: A meta-analysis. *Revista Paulista de Pediatria* 2020; **38**.

59 Pontoppidan M, Niss NK, Pejtersen JH, Julian MM, Væver MS. Parent report measures of infant and toddler social-emotional development: a systematic review. *Family Practice* 2017; **34**: 127-37.

60 Rah SS, Jung M, Lee K, Kang H, Jang S, Park J, Yoon JY, Hong SB. Systematic Review and Meta-Analysis: Real-World Accuracy of Children's Developmental Screening Tests. *Journal of the American Academy of Child & Adolescent Psychiatry* 2022. Article in Press.

61 Randall M, Egberts KJ, Samtani A, Scholten RJPM, Hooft L, Livingstone N, Sterling-Levis K, Woolfenden S, Williams K. Diagnostic tests for autism spectrum disorder (ASD) in preschool children. *Cochrane Database of Systematic Reviews* 2018; **2018**.

62 Romine CB, Lee D, Wolfe ME, Homack S, George C, Riccio CA. Wisconsin Card Sorting Test with children: A meta-analytic study of sensitivity and specificity. *Archives of Clinical Neuropsychology* 2004; **19**: 1027-41.

63 Ruffini C, Tarchi C, Morini M, Giuliano G, Pecini C. Tele-assessment of cognitive functions in children: A systematic review. *Child Neuropsychology* 2022; **28**: 709-45.

64 Salgado-Cacho JM, Moreno-Jimenez MDP, de Diego-Otero Y. Detection of Early Warning Signs in Autism Spectrum Disorders: A Systematic Review. *Children (Basel)* 2021; **8**.

65 Sánchez-García AB, Galindo-Villardón P, Nieto-Librero AB, Martín-Rodero H, Robins DL. Toddler Screening for Autism Spectrum Disorder: A Meta-Analysis of Diagnostic Accuracy. *Journal of Autism & Developmental Disorders* 2019; **49**: 1837-52.

66 Sansavini A, Favilla ME, Guasti MT, Marini A, Millepiedi S, Di Martino MV, Vecchi S, Battajon N, Bertolo L, Capirci O, Carretti B, Colatei MP, Frioni C, Marotta L, Massa S, Michelazzo L, Pecini C, Piazzalunga S, Pieretti M, Rinaldi P, Salvadorini R, Termine C, Zuccarini M, D’amico S, De Cagno AG, Levorato MC, Rossetto T, Lorusso ML. Developmental language disorder: Early predictors, age for the diagnosis, and diagnostic tools. A scoping review. *Brain Sciences* 2021; **11**.

67 Schwob S, Eddé L, Jacquin L, Leboulanger M, Picard M, Oliveira PR, Skoruppa K. Using Nonword Repetition to Identify Developmental Language Disorder in Monolingual and Bilingual Children: A Systematic Review and Meta-Analysis. *Journal of Speech, Language & Hearing Research* 2021; **64**: 3578-93.

68 Shahbazi M, Mirzakhani N. Assessment of Sensory Processing Characteristics in Children Between 0 and 14 Years of Age: A Systematic Review. *Iranian Journal of Child Neurology* 2021; **15**: 29-46.

69 Shahmahmood TM, Jalaie S, Soleymani Z, Haresabadi F, Nemati P. A systematic review on diagnostic procedures for specific language impairment: The sensitivity and specificity issues. *Journal of Research in Medical Sciences* 2016; **21**: 1-16.

70 Sim F, Thompson L, Marryat L, Ramparsad N, Wilson P. Predictive validity of preschool screening tools for language and behavioural difficulties: A PRISMA systematic review. *PLoS ONE [Electronic Resource]* 2019; **14**.

71 Slater LM, Hillier SL, Civetta LR. The clinimetric properties of performance-based gross motor tests used for children with developmental coordination disorder: a systematic review. *Pediatric Physical Therapy* 2010; **22**: 170-9.

72 Smith KG, Corkum P. Systematic review of measures used to diagnose attention-deficit/hyperactivity disorder in research on preschool children. *Topics in Early Childhood Special Education* 2007; **27**: 164-73.

73 So KKH, To CKS. Systematic Review and Meta-Analysis of Screening Tools for Language Disorder. *Frontiers in Pediatrics* 2022; **10**.

74 Soler N, Cordier R, Perkes IE, Dale RC, Bray P. Proxy-reported sensory measures for children and adolescents with neurodevelopmental disorders: A systematic review. *Developmental Medicine & Child Neurology* 2022; **30**: 30.

75 Song HQ, Lau PWC, Wang JJ. Investigation of the motor skills assessments of typically developing preschool children in China. *BMC Pediatrics* 2022; **22**.

76 Spittle AJ, Doyle LW, Boyd RN. A systematic review of the clinimetric properties of neuromotor assessments for preterm infants during the first year of life. *Developmental Medicine & Child Neurology* 2008; **50**: 254-66.

77 van Dokkum NH, Reijneveld SA, de Best JTBW, Hamoen M, Te Wierike SCM, Bos AF, de Kroon MLA. Criterion Validity and Applicability of Motor Screening Instruments in Children Aged 5–6 Years: A Systematic Review. *International Journal of Environmental Research and Public Health* 2022; **19**.

78 Velikonja T, Edbrooke‐Childs J, Calderon A, Sleed M, Brown A, Deighton J. The psychometric properties of the Ages & Stages Questionnaires for ages 2-2.5: a systematic review. *Child: Care, Health & Development* 2017; **43**: 1-17.

79 Wagner LV, Davids JR, Wagner LV, Davids JR. Assessment tools and classification systems used for the upper extremity in children with cerebral palsy. *Clinical Orthopaedics & Related Research®* 2012; **470**: 1257-71.

80 Wallen M, Stewart K. Upper limb function in everyday life of children with cerebral palsy: description and review of parent report measures. *Disability & Rehabilitation* 2015; **37**: 1353-61.

81 Warnick EM, Bracken MB, Kasl S. Screening efficiency of the child behavior checklist and strengths and difficulties questionnaire: A systematic review. *Child and Adolescent Mental Health* 2008; **13**: 140-7.

82 Wieckowski AT, Williams LN, Rando J, Lyall K, Robins DL. Sensitivity and Specificity of the Modified Checklist for Autism in Toddlers (Original and Revised): A Systematic Review and Meta-analysis. *Journal of the American Medical Association Pediatrics* 2023; **177**: 373-83.

83 Wong HS, Santhakumaran S, Cowan FM, Modi N. Developmental Assessments in Preterm Children: A Meta-analysis. *Pediatrics* 2016; **138**: 1-12.

84 Yin Foo R, Guppy M, Johnston LM. Intelligence assessments for children with cerebral palsy: a systematic review. *Developmental Medicine & Child Neurology* 2013; **55**: 911-8.

85 Yuen T, Penner M, Carter MT, Szatmari P, Ungar WJ. Assessing the accuracy of the Modified Checklist for Autism in Toddlers: a systematic review and meta-analysis. *Developmental Medicine & Child Neurology* 2018; **60**: 1093-100.

86 Heineman KR, Hadders-Algra M. Evaluation of neuromotor function in infancy-a systematic review of available methods. *Journal of developmental and behavioral pediatrics.* 2008; **29**: 315-23.
