# Supplementary material for: Clinical utility and psychometric properties of tools for early detection of developmental concerns and disability in young children: A scoping review
Source: Dev Med Child Neurol. 2024 Sep 16;67(3):286–306. doi: 10.1111/dmcn.16076 (PMC11794681; doi:10.1111/dmcn.16076)
Supplement: Supplementary file 6 — Figure S1: Flowchart. [file DMCN-67-286-s002.docx]

**Figure S1. PRISMA Flow chart for Systematic review search**

401 duplicates removed

1206 studies imported for screening

690 studies irrelevant

805 studies screened

30 studies excluded studies irrelevant

- 16 wrong outcomes
- 4 wrong patient populations
- 4 wrong study design
- 3 not psychometric study
- 2 unclear data
- 1 thesis

115 full text studies assessed for eligibility

86 systematic reviews included

6 clinical guidelines

132 papers

85 systematic reviews included from search

- 2 international clinical practice guidelines found from additional searching (CP, DCD)
- 4 clinical practice guidelines found in grey literature publications (ASD, ADHD, FASD, Victorian Early) Years: Communication)
- 133 papers searched on tools
- 1 systematic review found in search following reviewer feedback

67 tools included

(64 screening and assessment tools and

3 classification tools included)
